# Genetic evidence for malaria vectors of the *Anopheles sundaicus *complex in Sri Lanka with morphological characteristics attributed to *Anopheles subpictus *species B

**DOI:** 10.1186/1475-2875-9-343

**Published:** 2010-11-29

**Authors:** Sinnathamby N Surendran, Om P Singh, Pavillupillai J Jude, Ranjan Ramasamy

**Affiliations:** 1 Department of Zoology, Faculty of Science, University of Jaffna, Jaffna, Sri Lanka; 2 National Institute of Malaria Research, Sector 8, Dwarka, Delhi-110077, India; 3 Institute of Medicine, Universiti Brunei Darussalam, Gadong, Brunei Darussalam

## Abstract

**Background:**

*Anopheles subpictus sensu lato*, a widespread malaria vector in Asia, is reportedly composed of four sibling species A - D. Mosquitoes morphologically identified as belonging to the Subpictus complex were collected from different locations near the east coast of Sri Lanka, and specific ribosomal DNA sequences determined to validate their taxonomic status.

**Methods:**

*Anopheles subpictus s.l*. larvae and blood-fed adults were collected from different locations in the Eastern province and their sibling species status was determined based on published morphological characteristics. DNA sequences of the D3 domain of 28 S ribosomal DNA (rDNA) and the internal transcribed spacer -2 (ITS-2) of mosquitoes morphologically identified as *An. subpictus *sibling species A, B, C and D were determined.

**Results:**

Phylogenetic analysis based on D3 domain of rDNA resulted in two clades: one clade with mosquitoes identified as *An. subpictus *species A, C, D and some mosquitoes identified as species B, and another clade with a majority of mosquitoes identified as species B with D3 sequences that were identical to *Anopheles sundaicus *cytotype D. Analysis of ITS-2 sequences confirmed a close relationship between a majority of mosquitoes identified as *An. subpictus *B with members of the *An. sundaicus *complex and others identified as *An. subpictus *B with *An. subpictus s.l*.

**Conclusions:**

The study suggests that published morphological characteristics are not specific enough to identify some members of the Subpictus complex, particularly species B. The sequences of the ITS-2 and D3 domain of rDNA suggest that a majority that were identified morphologically as *An. subpictus *species B in the east coast of Sri Lanka, and some identified elsewhere in SE Asia as *An. subpictus s.l*., are in fact members of the Sundaicus complex based on genetic similarity to *An. sundaicus s.l*. In view of the well-known ability of *An. sundaicus s.l*. to breed in brackish and fresh water and its proven ability to transmit malaria in coastal areas of many Southeast Asian countries, the present findings have significant implications for malaria control in Sri Lanka and neighbouring countries.

## Background

*Anopheles culicifacies *species E is the major vector of malaria in Sri Lanka [1]. However *Anopheles subpictus s.l*. also plays a role in transmitting *Plasmodium vivax *and *Plasmodium falciparum *in many parts of Sri Lanka [1-4] as well as in Southeast Asia and India [5,6]. The taxon *An. subpictus *is reported to be a species complex comprising four members *viz*. species A, B, C and D in India [5,7]. The four members of this complex have been described as possessing characteristic paracentric fixed inversions on the X-chromosome *viz*. species A (X+^a^, +^b^), species B (Xa, b), species C (Xa, +^b^) and species D (X+^a^, b) [5,7]. Additionally, stage-specific morphometric characteristics e.g. the number of ridges in egg floats, larval mesothoracic seta 4, pupal setae and ornamentation of the palpi of adult females, have been reported to be useful for differentiating *An. subpictus *sibling species in field studies [7]. Initial studies based on a single inversion in the X chromosome suggested the presence of *An. subpictus *species A and B in Sri Lanka [8]. Subsequent studies based on the numbers of egg ridges indicated the presence of all four sibling species in the country [9]. Members of the complex differ in breeding site and feeding preference and seasonal abundance. The bio-ecological differences between members of the *An. subpictus *complex in Sri Lanka [1] and elsewhere [5] have been recently reviewed.

Although all four species of *An. subpictus *are reported to breed in fresh and brackish waters in India, species B is mainly reported to breed in brackish waters [5,10] and predominate in coastal areas [7,11]. The coastal populations of the Subpictus complex have been particularly incriminated as malaria vectors in India [12,13] and Sri Lanka [8]. *An. subpictus*, including species B, has previously been detected in Eastern Sri Lanka [14,15]. *An. subpictus *species B has been specifically implicated in transmitting malaria in the coastal areas of the Puttalam district in the west coast of Sri Lanka [8].

The predominantly brackish water breeding *Anopheles sundaicus s.l*. is a major vector of malaria in coastal areas of Southeast Asia and Bangladesh [16,17]. *Anopheles sundaicus s.l*. was also an important vector of malaria along the east coast of India, including the state of Tamil Nadu (situated in close proximity to Sri Lanka) until DDT spraying largely eliminated it from mainland India, confining it more to the Andaman and Nicobar islands [17,18]. *Anopheles sundaicus s.l*. has never previously been detected in Sri Lanka. The taxon *An. sundaicus *is reported to exist as a species complex comprising four cytological forms (A-D) and four molecular forms namely *An. sundaicus s.s*., *Anopheles epiroticus*, *An. sundaicus *cytotype D and *An. sundaicus *E [17-19] that occupy fresh and brackish water habitats.

*Anopheles vagus *is a species related to *An. subpictus s.l*. and *An. sundaicus s.l*. that is classified under the same subgenus *Cellia *and series Pyretophorous. It has been implicated as a secondary malaria vector elsewhere in the Indian subcontinent [20] and is present in Sri Lanka [3,21].

Morphological properties are widely used to identify and classify mosquito species in field studies because large numbers of samples can be processed without the need for specialized and expensive equipment, which is a common drawback in resource-limited laboratories. However genetic characterization provides the more definitive identification. DNA sequences that give rise to ribosomal RNA (rRNA) are widely used for phylogenetic analysis of closely related organisms including mosquitoes [22,23]. This is because eukaryotic organisms contain many tandemly repeated copies of rRNA genes that tend to homogenize nucleotide sequences within panmictic species but diverge in sequence between reproductively isolated species. In this context, the expansion loops of 28 S ribosomal RNA, such as the D3 domain, show significantly greater variability between species than core structures. Even greater variability due to more rapid evolution is found in the non-coding internally transcribed spacer (ITS) sequences such as ITS-2, which lies between the 5.8 S and 28 S RNA genes. The D3 and ITS-2 sequences of mosquitoes morphologically identified as *An. subpictus *species B collected from different coastal locations in the Eastern province of Sri Lanka, and the importance of the findings for understanding malaria transmission in Sri Lanka and elsewhere in Asia are reported here.

## Methods

### Mosquito collection and identification of sibling species of *An. subpictus*

**Figure 1:**
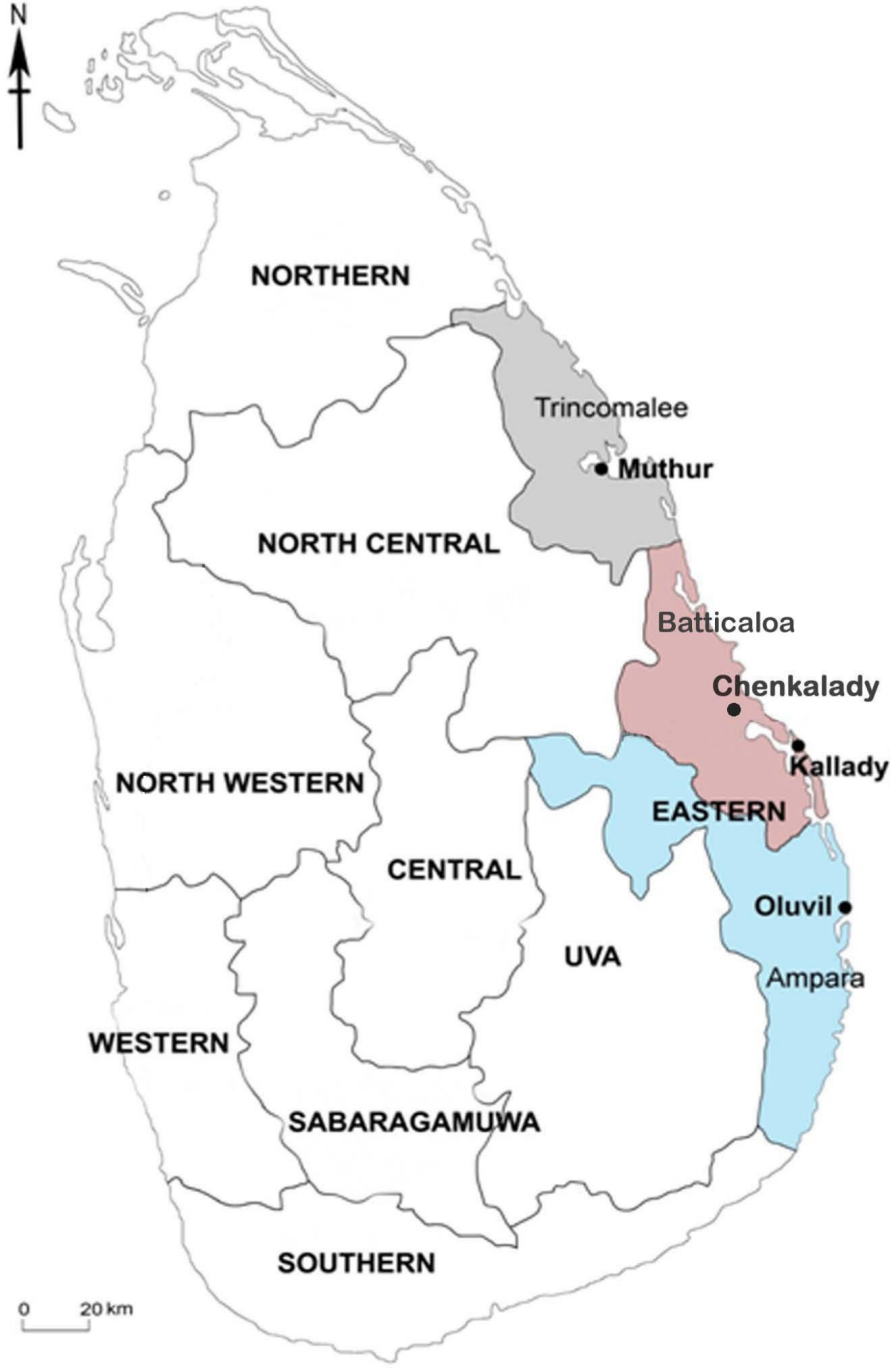
**Mosquito collection sites in the districts of Ampara, Batticaloa and Trincomalee of the Eastern province (coloured areas) of Sri Lanka**.

Blood-fed adult anopheline mosquitoes were collected in January and August 2009 from four collection sites in three locations *viz*. Oluvil (one site within ≈2 km from the coast; 7° 21" 44.41' N: 81° 50" 42.68' E), Chenkalady (one site ≈ 4 km from the coast; 7° 33" 54.93' N: 81° 36" 44.61' E) and Muthur (two sites: an inland area ≈ 8 km from the coast: 8° 25" 29.48' N: 81° 16" 00.97' E; and another < 1 km from the coast: 8° 27" 23.90' N: 81° 15" 33.91' E) in the respective districts of Ampara, Batticaloa and Trincomalee of the Eastern province of Sri Lanka (Figure 1) using cattle baited hut and cattle baited net collections. Larval forms were also collected from a brackish water body in Kallady (< 1 km from the coast and contiguous with the Batticaloa lagoon; 7°43'07.16" N; 81°42'31.28" E) (Figure 1) as previously described [14].

The collected adults were brought to the Zoology laboratory of the Eastern University and identified as *An. subpictus s.l*. using published keys [6,24]. Prominent characteristics used for identification were wings with contrasting pale and dark scales, leading margin of wing with at least 4 separate dark areas characteristic of Subgenus *Cellia*, hind tarsomers 3, 4 and 5 not entirely white scaled, femur and tibia not speckled, foretarsi with broad pale bands crossing joints, palps with preapical dark band equal to or slightly less than the length of apical pale band. Larvae collected at Kallady were screened for reported characteristics of mesothoracic seta 4 (seta 4 M; bifurcate for *An. subpictus *species A and B but the latter with shorter and thinner branches) for identifying sibling species of the Subpictus complex [7], and reared in the laboratory until they became adults. Facilities and expertise were not available for determining polytene chromosome banding patterns during this investigation and therefore only morphological characteristics were used in identification. Identified blood-fed females were maintained individually and single female F1 progenies were raised as described previously [25]. Sibling species status of females laying eggs was determined through the reported number of ridges in the floats of egg that characterize the different species i.e. species A, 31-36; species B, 16-20; species C, 25-29 and species D, 21-24 [7]. Five to ten eggs from each female were placed on a clean microscopic slide and the number of ridges on floats was counted under a light microscope (x4, Olympus). Adult emerging from identified isofemale progenies were used to examine the ornamentation of wing and palpi. Adult F1 progenies of identified sibling species A, B, C and D were coded and preserved in microcentrifuge tubes containing silica gel plugged with a small piece of cotton wool for molecular studies. Similarly adults that emerged from collected larvae were also preserved separately for molecular characterization.

### PCR amplification, DNA sequencing and phylogenetic analysis

The DNA of individual mosquitoes was isolated following the method described by Livak [26]. The ITS-2 region of the rDNA was amplified using primers ITS-2A (5' - TGT GAA CTG CAG GAC ACA T- 3') and ITS-2B (5'- TAT GCT TAA ATT CAG GGG GT - 3') [27]. The D3 domain of 28 S rDNA was amplified using D3A (5'-GAC CCG TCT TGA AAC ACG GA-3') and D3B (5'- TCG GAA GGA ACC AGT TAC TA - 3') primers [28]. For each PCR assay, 50 μl PCR reaction mixture was used. Each mixture contained 0.50 μM of each primer, 200 μM of each dNTP, 1.5 mM of MgCl_2 _and 1.25 unit of taq polymerase (AmpliTaq Gold, Applied Biosystem). The PCR conditions for both PCRs were an initial denaturation at 95°C for 5 min followed by 35 cycles of 95°C for 30 s, 55°C for 30 s, and 72°C for 45 s followed by a final extension at 72°C for 7 min. The PCR products that were successfully amplified were then purified using the Quiaquik PCR Purification Kit (Qiagen, California, USA) to remove unincorporated primers and dNTPs prior to sequencing. The purified PCR amplicons were sent to M/s Macrogen Inc., South Korea, for sequencing in both the forward and reverse directions.

The derived sequences obtained were then analysed together with other representative sequences obtained from GenBank using ClustalW. Neighbour-Joining phylogeny tree was constructed with Bootstrap values from 500 replicates using the MEGA software [29]. Details of the collection sites of different Sri Lankan *An. subpictus *specimens from the present study and that of *An. vagus *and other members of the Subpictus and Sundaicus complexes obtained from GenBank and used for phylogenetic analysis of DNA sequences are also summarized in Table 1.

**Table 1:** Collection sites of An. subpictus, An. sundaicus and An. vagus specimens used in the phylogenetic analyses

Specimen	Collection site	D3 accession number	ITS-2 accession number
*An. sundaicus *cytotype D	Nicobar island, India	AY691512	

*An. sundaicus *cytotype D	Nicobar island, India	AY691513	

*An. sundaicus *cytotype D	Andaman island, India	AY691516	

*An. subpictus *B (LK-B-2, 3, 5, 25, 26, 28)	Kallady, Sri Lanka	Present study	

*An. subpictus *B (LK-B- 13-15, 17)	Chenkalady, Sri Lanka	Present study	

*An. subpictus *B (LK-B-18-21)	Kalmunai, Sri Lanka	Present study	

*An. subpictus *B (LK-B-6, 7, 9, 10, 11, 16, 22, 23, 24, 31)	Kallady, Sri Lanka	Present study	Present study

*An. subpictus *B (LK-B-1, 4, 8, 12,)	Kallady, Sri Lanka	Present study	

*An. subpictus *B (LK-B- 27, 29, 30)	Kallady, Sri Lanka	Present study	

*An. subpictus *A (LK-A-1-5)	Chenkalady, Sri Lanka	Present study	

*An. subpictus *C (LK-C- 1-5)	Chenkalady, Sri Lanka	Present study	

*An. subpictus *D (LK-D-1-5)	Chenkalady, Sri Lanka	Present study	

*An. subpictus s.l*.	Monaragala, Sri Lanka		GQ870337.1

*An. subpictus s.l*.	Monaragala, Sri Lanka		GQ870336.1

*An. subpictus s.l*.	Inland area, Sri Lanka		AF406615.1

*An. subpictus s.l*.	Coastal area, Sri Lanka		AF406616.2

*An. subpictus s.l*.	Inland area, Sri Lanka		AF406614.1

*An. subpictus s.l*.	Inland area, Sri Lanka		AF406613.1

*An. subpictus s.l*.	Coastal area, Sri Lanka		AY049004.1

*An. subpictus s.l*.	Punjab, India		EF601870.1

*An. subpictus s.l*.	Punjab, India		EF601869.1

*An. subpictus s.l*.	Punjab, India		EF601868.1

*An. subpictus s.l*.	Java, Indonesia		GQ870337.1

*An. subpictus s.l*.	Rakhine, Myanmar		GQ870334.1

*An. subpictus s.l*.	Phang Nya, Thailand		GQ870333.1

*An. subpictus s.l*.	Ninh Binh, Vietnam		GQ870330.1

*An. subpictus s.l*.	Kampot, Cambodia		GQ870329.1

*An. subpictus s.l*.	Flores, Indonesia		GQ870328.1

*An. subpictus s.l*.	Kampot, Cambodia		GQ870326.1

*An. subpictus s.l*.	Can Gio, Vietnam		GQ870325.1

*An. epiroticus*	Can Gio, Vietnam		AY662445.1

*An. sundaicus *cytotype D	Andaman islands, India		AY691517.1

*An. sundaicus s.l*.	Lundu, Malaysia		AF369562.1

*An. sundaicus s.l*.	Trat Province, Thailand		AF469857.1

*An. sundaicus s.l*.	Sarawak, Malaysia		AY662258.1

*An. sundaicus s.l*.	Timor-Leste		GQ480826.1

*An. vagus E*	Timor-Leste		GQ480823.1

*An. vagus*	Hainan island, China	FJ457630	

*An. vagus*	Hainan island, China		FJ457631.1

*An. vagus*	Indonesia		FJ654649.1

## Results

### Identification of *An. subpictus *sibling species and morphological variations in sibling species B

A total of 246 blood-fed *An. subpictus s.l*. were collected from the four field sites in this study and based on number of ridges on the laid eggs, 2, 152, 11 and 6 females were tentatively characterized as belonging to species A, B, C and D respectively. Different combinations of variant patterns in wing ornamentation (variation in the costal pre-humeral dark spot and pre-apical dark spot) and palpal ornamentation (relative lengths of the apical pale and sub-apical dark bands) in those mosquitoes identified as *An. subpictus *species B were observed. In the larval collection from Kallady, all 54 adults that emerged from collected larvae were tentatively identified as species B, based on morphological characteristics of larvae and adults.

### Phylogenetic analysis based on D3 rRNA

Construction of a phylogenetic tree based on D3 rDNA sequences (Additional file 1) resulted in three stable clades (bootstrap values ≥ 95%). The D3 rDNA phylogenetic tree analysis is presented in Additional file 2. One clade consisted of 14 mosquitoes identified as *An. subpictus *species A, C, or D and seven identified as species B collected in the present study (LK-B1, 4, 8, 12, 27, 29 and 30) and is termed the *An. subpictus *clade. All members in this clade possessed identical D3 sequences.

A second clade, termed the *An. sundaicus *clade, contained 22 mosquitoes identified as *An. subpictus *species B (LK- B2, 3, 5, 7, 9-11, 13-26, 28) based on the same morphological characteristics as the species B members falling into the *An. subpictus *clade. The D3 rDNA sequences of all members identified as *An. subpictus *species B in the *An. sundaicus *clade (Additional file 2) were identical to those of *An. sundaicus *cytotype D samples (AY691512, AY691513 and AY691516) collected from the Andaman and Nicobar islands in an unrelated study [30]. The differences between the *An. subpictus *and *An. sundaicus *clades were caused by six single nucleotide and two trinucleotide changes and one insertion/deletion within the analysed sequences.

An *An. vagus *voucher specimen from China (FJ457630.1) formed a third clade that was more closely related to the *An. subpictus *clade than to the *An. sundaicus *clade (Additional file 2).

### Phylogenetic analysis based on ITS-2 rRNA

**Figure 2:**
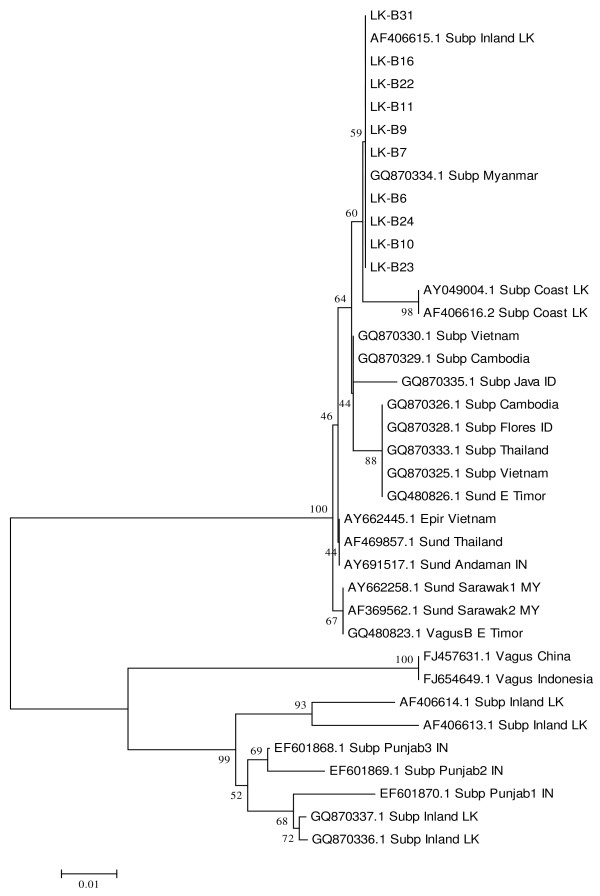
**Phylogenetic analysis based on ITS-2 of the rDNA**. LK- B series refer to individual specimens collected in the study that were initially morphologically characterized as belonging to *An. subpictus *species B. Other sequences of *An. sundaicus s.l*. (Sund), *An subpictus *(Subp) and *An. vagus *(Vagus) from different locations in countries indicated by their standard two letter abbreviations, were obtained from GenBank.

Sequence analysis of the ITS-2 sequences of the presumed *An. subpictus *species B collected in the Eastern province in this study and other available GenBank sequences for *An. subpictus s.l*. and *An. sundaicus s.l*. (Additional file 3) were also carried out. The results showed that all species B-type specimens from this study (LK-B series) that formed the *An. sundaicus *clade with members of Sundaicus complex based on their D3 sequence (LK-B7, 9-11, 16, and 22-24) also stably clustered with *An. sundaicus s. l*. (AF 369562 and AF 662258) and *An. epiroticus *based on ITS-2 sequences (Figure 2). This clade is termed the *An. sundaicus *clade. The only two additional *An subpictus *B-like specimens in this study whose ITS-2 sequences were determined (LK-B6 and 31) were also found to fall into this *An. sundaicus *clade. Several specimens identified as *An. subpictus s.l*. from many other locations in Southeast Asia also grouped with the *An. sundaicus *clade on analysis of the ITS-2 sequences. These included one specimen (AF406615.1) isolated from an unspecified inland site in Sri Lanka and two others collected from unspecified coastal areas of Sri Lanka (AY049004.1 and AF406616.2). The *An. sundaicus *clade also included specimens classified as *An. subpictus s.l*. from Myanmar, Vietnam, Cambodia, Indonesia and Thailand. The ITS-2 sequences were identical in all of the *An. subpictus *species B specimens collected in this study, the LK-B series, from different sites in Eastern Sri Lanka (Additional file 3). These were also identical to the ITS-2 sequence in *An. subpictus s.l*. from Myanmar (GQ870334.1), differed from *An. subpictus s.l *(AF406615.1) in the absence of a single insertion present in the latter, and differed from the two other independent *An. subpictus s.l*. isolates from Sri Lanka (AY049004.1 and AF406616.2) in three nucleotide changes that were common to the latter (Additional file 3).

A second ITS-2 clade in this analysis, termed the *An. subpictus *clade, was composed of inland populations of *An. subpictus s.l*. from Punjab, India and Sri Lanka isolated by independent researchers and whose sibling status had not been determined. These could conceivably have all belonged to species A, C or D of Subpictus complex. The ITS-2 sequences from specimens identified as *An. subpictus *species A, C or D that formed the *An. subpictus *complex on analysis of the D3 sequences were not available. However ITS-2 sequences from two different specimens (LK-A21 and LK-A22) identified as *An. subpictus *species A in the present collection were identical to those from two *An. subpictus s.l*. specimens collected from Southern Sri Lanka by others (GQ870337.1 and GQ870336.1) and that clearly fell into the *An. subpictus *ITS-2 clade on phylogenetic analysis (Figure 2 and Additional file 3).

Two of the available ITS-2 sequences from *An. vagus *(FJ457631.1, a voucher specimen from Hainan island in China and FJ654649 from Indonesia) formed a separate clade that was more closely related to the *An. subpictus *clade than the *An. sundaicus *clade.

While the three major clades were firmly established with bootstrap values ≥ 99%, some of the internal branches within the *An. subpictus *and *An. sundaicus *clades had bootstrap values < 70% indicating a lower degree of robustness in the proposed separation (Figure 2).

## Discussion

The Subpictus complex, the Sundaicus complex, *An. vagus *and the recently described *An. pseudosundaicus *[31] are closely related taxons. They have been reported to differ in the ornamentation of wings and palpi, properties that are commonly used in identification keys of Anopheline mosquitoes [31]. However only the taxons *An. subpictus *and *An. sundaicus *are known to exist as species complexes. Morphological characters that are commonly used to distinguish the four taxons are summarized in Additional file 4. A prominent morphological character that differentiates adult *An. sundaicus s.l*. from *An. subpictus s.l*. and *An. vagus *is reported to be the speckling of the femur and tibia of the legs of *An. sundaicus s.l*. [6,31]. Specimens identified as *An. subpictus *species B in the present study did not show prominent speckling in their femur and tibia. There is an overlap in the reported number of egg ridges that distinguish *An. subpictus *species B and *An. sundaicus s.l*. [6]. In *An. subpictus *species B the number of egg ridges vary from 16-20 while it is reported to be about 20 for *An. sundaicus s.l*. [6]. All four cytotypes (A - D) of the Sundaicus complex can be differentiated by examining the distribution of heterochromatin blocks [32,33]. On the other hand, X- chromosomal inversion polymorphism and egg morphology are generally used to discriminate members of the Subpictus complex [5]. The existence of adult (length variation of apical pale band and subapical dark band of female palpi), larval (characteristic of seta 4 M) and pupal (setae of 6, 7, 9-I) morphological variations among members of the Subpictus complex have been reported (Additional file 4) [7,34].

Importantly, genetic analysis of specimens morphologically identified as *An. subpictus *B in this study showed that a majority are probably members of the Sundaicus complex distinct from species A, C and D of the *An. subpictus *complex as well as *An. vagus*. This group of *An. sundaicus s.l*. - like mosquitoes were probably incorrectly identified as *An. subpictus *B based on morphological characteristics. A minority of specimens identified as *An. subpictus *species B in the present study based on the number of ridges in egg floats or ornamentation, and which were genetically related based on D3 sequences to species A, C and D, may actually be species A, C or D of the Subpictus complex. This is because the reported differences between Indian species B and D on the number of egg ridges and between Indian species B, C and D in adult ornamentation are small [7]. The morphological differences between *An. subpictus *species A - D reported to exist in India may also not be altogether applicable to Sri Lankan *An. subpictus *species due to morphological changes consequent to isolation in an island. There is an alternative possibility that the specimens that were morphologically like the Indian *An. subpictus *species B but with D3 sequences related to species A, C and D might belong to the archetypical *An. subpictus *species B category originally identified in India through differential X-chromosome morphology [5,7]. Further chromosomal, molecular and morphological studies are needed to clarify these possibilities.

The genetic data is consistent with a panmictic population of *An. sundaicus *sibling species being present in the Eastern province of Sri Lanka. The identity of the ITS-2 sequence of this species with that from a specimen identified as *An. subpictus s.l*. from Myanmar suggests that the latter may belong to the same species. This is consistent with the relative coastal proximity and historically close maritime contacts between Eastern Sri Lanka and Myanmar. One other hand, *An. subpictus s.l *specimen collected previously from elsewhere in Sri Lanka differed from the *An. sundaicus *LK-B series sequence in a single insertion while two other specimens showed three nucleotide changes. Hence it is possible that there is some reproductive isolation among *An. sundaicus s.l*. species in Sri Lanka that has led to differences in the ITS-2 sequences. In this context it will be important to examine the D3 and ITS-2 sequences of mosquitoes previously identified as *An. subpictus *species B in the Puttalam district in the West coast of Sri Lanka where they are reported to be the major vector of coastal malaria [8]. The present findings also suggest that the two genotypes, *An. subpictus s.l*. and *An. sundaicus s.l*. may occur sympatrically in habitats close to the coast.

There is also the indication from available ITS-2 data that members of the Sundaicus complex may have been mistakenly identified as *An. subpictus s.l*. in other parts of Southeast Asia. Compatible conclusions were reached with a cryptic species tentatively termed *An. vagus *genotype B in Timor Leste based on morphological characterization [35]. The *An. vagus *genotype B, was morphologically similar in the absence of speckling in its femur and tibia to *An. vagus *and *An. subpictus s.l*. than to *An. sundaicus s.l*. However it is clustered with *An. sundaicus s.l*. as well as some specimens identified morphologically as *An. subpictus s.l*. populations of Sri Lanka on phylogenetic analysis of its ITS-2 sequence [35]. Indeed it is possible that most, if not all, of the reported *An. subpictus *species B like-mosquitoes in Sri Lanka, and perhaps elsewhere in Southeast Asia, are in fact members of the *An. sundaicus *complex. Detailed studies of chromosome inversions and molecular genotyping of Asian *An. subpictus *species B-like mosquitoes is needed to establish whether this is indeed the case.

Morphological characterization of mosquitoes provides a simple, inexpensive and readily applicable way of identifying many malaria vectors in the field. However, there are clearly limitations with using morphological characteristics alone to differentiate anopheline mosquitoes where they exist sympatically as species complexes or as closely related species. In the context of malaria transmission, further molecular characterization utilizing additional mitochondrial genes such as cytochrome oxidase subunits (CO) and cytochrome B (Cyt-b) are additionally needed to characterize the presence of sibling species in Subpictus complex and Sundaicus complex in Asia. This will aid the development of more robust morphology-based or DNA-based identification methods that would be suitable for field-based applications.

## Conclusions

Sequence analysis of ITS-2 and 28 S RNA D3 domains of rDNA show that a majority of specimens morphologically identified as *An. subpictus *species B in Eastern Sri Lanka are in fact members of the Sundaicus complex. The existing morphological variations and their use for identifying closely related anopheline mosquitoes, especially when they exist as species complexes, are imprecise and need to be replaced with DNA sequence-based techniques. The results also suggest that molecular techniques for classifying the relationships between members of the Sundaicus and Subpictus complexes are particularly important in South and Southeast Asia where members of the Sundaicus complex may have a hitherto unappreciated geographical spread helped possibly by long-existing maritime links between the nations of the region. Members of the Sundaicus complex, that are well known malaria vectors in coastal areas, pose the risk of increasing malaria transmission with a rise in sea levels due to global warming and expanding aquaculture. These findings have a direct bearing on the continued development of effective malaria vector control or elimination programmes in Sri Lanka and elsewhere in Asia.

## Competing interests

The authors declare that they have no competing interests.

## Authors' contributions

SNS and RR conceived the study. PJJ and SNS performed all the field and laboratory studies. OPS did sequence analysis and RR performed phylogenetic analysis. RR and SNS wrote the manuscript. All authors read and approved the final manuscript.

## Supplementary Material

Additional file 1 **D3 sequences used for phylogenetic analysis**. Phylogenetic analysis is based on the D3 region of 28 S rDNA sequences. LK- A, B, C and D series that refer to individual specimens collected in the present study in Sri Lanka that were initially morphologically characterized as belonging to *An. subpictus *species A, B, C and D respectively. The sequences of *An. sundaicus *(Sund) from Andaman and Nicobar islands of India (IN) and *An. vagus *(Vagus) from China were obtained from GenBank. Click here for file

Additional File 2 **Phylogenetic analysis based on the D3 region of 28 S rDNA sequences**. The analysis is based on LK-A, B, C and D series that were individual specimens collected in the study that were initially characterized as belonging to *An. subpictus *A, B, C and D respectively. The sequences of *An. sundaicus *(Sund) from Andaman and Nicobar silands of India (IN) and *An. vagus *(Vagus) from China were obtained from GenBank. Click here for file

Additional File 3 **ITS-2 sequences used for phylogenetic analysis**. Sequences of the ITS-2 region of rDNA used for phylogenetic analysis. LK- B series refer to individual specimens collected in the study that were initially morphologically characterized as belonging to *An. subpictus *species B. LK-A series refers to those similarly characterized as *An, subpictus *species A. Other sequences of *An. sundaicus s.l*. (Sund), *An subpictus *(Subp) and *An. vagus *(Vagus) from different locations in countries indicated by their standard two letter abbreviations, were obtained from GenBank. Click here for file

Additional File 4 **Morphological differentiation of *An. sundaicus*, *An. vagus*, *An. pseudosundaicus *and *An. subpictus***. Reported morphological characteristics that differentiate *An. sundaicus*, *An. vagus*, *An. pseudosundaicus *and *An. subpictus *populations and their distribution among sibling species of the Subpictus Complex Click here for file
