# Supplementary material for: Genetic evidence for malaria vectors of the Anopheles sundaicus complex in Sri Lanka with morphological characteristics attributed to Anopheles subpictus species B
Source: Malar J. 2010 Nov 29;9:343. doi: 10.1186/1475-2875-9-343 (PMC3009661; doi:10.1186/1475-2875-9-343)
Supplement: Additional file 1 — D3 sequences used for phylogenetic analysis. Phylogenetic analysis is based on the D3 region of 28 S rDNA sequences. LK- A, B, C and D series that refer to individual specimens collected in the present study in Sri Lanka that were initially morphologically characterized as belonging to An. subpictus species A, B, C and D respectively. The sequences of An. sundaicus (Sund) from Andaman and Nicobar islands of India (IN) and An. vagus (Vagus) from China were obtained from GenBank. [file 1475-2875-9-343-S1.DOC]

**D3 sequences used for phylogenetic analysis**

>LK-A1

TGGGACCGGTGCCACCTTCGGGTGGTGCCGCTCAATCGAACACCCATAGGCGAAGACAACTCGAAGACGCATGTCACGGGATTACGGGTTCGGCATTGGCGCAAGCCTACGTCGGATCCCTCCATCCCAGGGTGCCCCGATACGGGTGGGAGCGGGCCTCCGGGCGTCGTTCTACCCAGCGGGCATACCCCGAGTGTGCAGGATGCGACCCGAAAGATGGTGAACTATGCCTGATCAGGTTGAAGTCAGGGGAAACCCTGATGGAGGACCGAAGCAATTCTGACGTGCAAATCGATTG

>LK-A2

TGGGACCGGTGCCACCTTCGGGTGGTGCCGCTCAATCGAACACCCATAGGCGAAGACAACTCGAAGACGCATGTCACGGGATTACGGGTTCGGCATTGGCGCAAGCCTACGTCGGATCCCTCCATCCCAGGGTGCCCCGATACGGGTGGGAGCGGGCCTCCGGGCGTCGTTCTACCCAGCGGGCATACCCCGAGTGTGCAGGATGCGACCCGAAAGATGGTGAACTATGCCTGATCAGGTTGAAGTCAGGGGAAACCCTGATGGAGGACCGAAGCAATTCTGACGTGCAAATCGATTG

>LK-A3

TGGGACCGGTGCCACCTTCGGGTGGTGCCGCTCAATCGAACACCCATAGGCGAAGACAACTCGAAGACGCATGTCACGGGATTACGGGTTCGGCATTGGCGCAAGCCTACGTCGGATCCCTCCATCCCAGGGTGCCCCGATACGGGTGGGAGCGGGCCTCCGGGCGTCGTTCTACCCAGCGGGCATACCCCGAGTGTGCAGGATGCGACCCGAAAGATGGTGAACTATGCCTGATCAGGTTGAAGTCAGGGGAAACCCTGATGGAGGACCGAAGCAATTCTGACGTGCAAATCGATTG

>LK-A4

TGGGACCGGTGCCACCTTCGGGTGGTGCCGCTCAATCGAACACCCATAGGCGAAGACAACTCGAAGACGCATGTCACGGGATTACGGGTTCGGCATTGGCGCAAGCCTACGTCGGATCCCTCCATCCCAGGGTGCCCCGATACGGGTGGGAGCGGGCCTCCGGGCGTCGTTCTACCCAGCGGGCATACCCCGAGTGTGCAGGATGCGACCCGAAAGATGGTGAACTATGCCTGATCAGGTTGAAGTCAGGGGAAACCCTGATGGAGGACCGAAGCAATTCTGACGTGCAAATCGATTG

>LK-A5

TGGGACCGGTGCCACCTTCGGGTGGTGCCGCTCAATCGAACACCCATAGGCGAAGACAACTCGAAGACGCATGTCACGGGATTACGGGTTCGGCATTGGCGCAAGCCTACGTCGGATCCCTCCATCCCAGGGTGCCCCGATACGGGTGGGAGCGGGCCTCCGGGCGTCGTTCTACCCAGCGGGCATACCCCGAGTGTGCAGGATGCGACCCGAAAGATGGTGAACTATGCCTGATCAGGTTGAAGTCAGGGGAAACCCTGATGGAGGACCGAAGCAATTCTGACGTGCAAATCGATTG

>LK-B1

TGGGACCGGTGCCACCTTCGGGTGGTGCCGCTCAATCGAACACCCATAGGCGAAGACAACTCGAAGACGCATGTCACGGGATTACGGGTTCGGCATTGGCGCAAGCCTACGTCGGATCCCTCCATCCCAGGGTGCCCCGATACGGGTGGGAGCGGGCCTCCGGGCGTCGTTCTACCCAGCGGGCATACCCCGAGTGTGCAGGATGCGACCCGAAAGATGGTGAACTATGCCTGATCAGGTTGAAGTCAGGGGAAACCCTGATGGAGGACCGAAGCAATTCTGACGTGCAAATCGATTG

>LK-B2

TGGGACCGATACCACCGGTGTGTGGTGTCGCTCAATCGAACACCCATAGGCGAAGACAACTCGATGACGTTCGTCACGGGATTACGGGTTCGGCAT-GGCGCAAGCCTTCGTCGGATCCCTCCATCCCAGGGTGCCCCGATACGGGTGGGAGCGGGCCTCCGGGCGTCGTTCTACCCAGCGGGCATACCCCGAGTGTGCAGGATGCGACCCGAAAGATGGTGAACTATGCCTGATCAGGTTGAAGTCAGGGGAAACCCTGATGGAGGACCGAAGCAATTCTGACGTGCAAATCGATTG

>LK-B3

TGGGACCGATACCACCGGTGTGTGGTGTCGCTCAATCGAACACCCATAGGCGAAGACAACTCGATGACGTTCGTCACGGGATTACGGGTTCGGCAT-GGCGCAAGCCTTCGTCGGATCCCTCCATCCCAGGGTGCCCCGATACGGGTGGGAGCGGGCCTCCGGGCGTCGTTCTACCCAGCGGGCATACCCCGAGTGTGCAGGATGCGACCCGAAAGATGGTGAACTATGCCTGATCAGGTTGAAGTCAGGGGAAACCCTGATGGAGGACCGAAGCAATTCTGACGTGCAAATCGATTG

>LK-B4

TGGGACCGGTGCCACCTTCGGGTGGTGCCGCTCAATCGAACACCCATAGGCGAAGACAACTCGAAGACGCATGTCACGGGATTACGGGTTCGGCATTGGCGCAAGCCTACGTCGGATCCCTCCATCCCAGGGTGCCCCGATACGGGTGGGAGCGGGCCTCCGGGCGTCGTTCTACCCAGCGGGCATACCCCGAGTGTGCAGGATGCGACCCGAAAGATGGTGAACTATGCCTGATCAGGTTGAAGTCAGGGGAAACCCTGATGGAGGACCGAAGCAATTCTGACGTGCAAATCGATTG

>LK-B5

TGGGACCGATACCACCGGTGTGTGGTGTCGCTCAATCGAACACCCATAGGCGAAGACAACTCGATGACGTTCGTCACGGGATTACGGGTTCGGCAT-GGCGCAAGCCTTCGTCGGATCCCTCCATCCCAGGGTGCCCCGATACGGGTGGGAGCGGGCCTCCGGGCGTCGTTCTACCCAGCGGGCATACCCCGAGTGTGCAGGATGCGACCCGAAAGATGGTGAACTATGCCTGATCAGGTTGAAGTCAGGGGAAACCCTGATGGAGGACCGAAGCAATTCTGACGTGCAAATCGATTG

>LK-B7

TGGGACCGATACCACCGGTGTGTGGTGTCGCTCAATCGAACACCCATAGGCGAAGACAACTCGATGACGTTCGTCACGGGATTACGGGTTCGGCAT-GGCGCAAGCCTTCGTCGGATCCCTCCATCCCAGGGTGCCCCGATACGGGTGGGAGCGGGCCTCCGGGCGTCGTTCTACCCAGCGGGCATACCCCGAGTGTGCAGGATGCGACCCGAAAGATGGTGAACTATGCCTGATCAGGTTGAAGTCAGGGGAAACCCTGATGGAGGACCGAAGCAATTCTGACGTGCAAATCGATTG

>LK-B8

TGGGACCGGTGCCACCTTCGGGTGGTGCCGCTCAATCGAACACCCATAGGCGAAGACAACTCGAAGACGCATGTCACGGGATTACGGGTTCGGCATTGGCGCAAGCCTACGTCGGATCCCTCCATCCCAGGGTGCCCCGATACGGGTGGGAGCGGGCCTCCGGGCGTCGTTCTACCCAGCGGGCATACCCCGAGTGTGCAGGATGCGACCCGAAAGATGGTGAACTATGCCTGATCAGGTTGAAGTCAGGGGAAACCCTGATGGAGGACCGAAGCAATTCTGACGTGCAAATCGATTG

>LK-B9

TGGGACCGATACCACCGGTGTGTGGTGTCGCTCAATCGAACACCCATAGGCGAAGACAACTCGATGACGTTCGTCACGGGATTACGGGTTCGGCAT-GGCGCAAGCCTTCGTCGGATCCCTCCATCCCAGGGTGCCCCGATACGGGTGGGAGCGGGCCTCCGGGCGTCGTTCTACCCAGCGGGCATACCCCGAGTGTGCAGGATGCGACCCGAAAGATGGTGAACTATGCCTGATCAGGTTGAAGTCAGGGGAAACCCTGATGGAGGACCGAAGCAATTCTGACGTGCAAATCGATTG

>LK-B10

TGGGACCGATACCACCGGTGTGTGGTGTCGCTCAATCGAACACCCATAGGCGAAGACAACTCGATGACGTTCGTCACGGGATTACGGGTTCGGCAT-GGCGCAAGCCTTCGTCGGATCCCTCCATCCCAGGGTGCCCCGATACGGGTGGGAGCGGGCCTCCGGGCGTCGTTCTACCCAGCGGGCATACCCCGAGTGTGCAGGATGCGACCCGAAAGATGGTGAACTATGCCTGATCAGGTTGAAGTCAGGGGAAACCCTGATGGAGGACCGAAGCAATTCTGACGTGCAAATCGATTG

>LK-B11

TGGGACCGATACCACCGGTGTGTGGTGTCGCTCAATCGAACACCCATAGGCGAAGACAACTCGATGACGTTCGTCACGGGATTACGGGTTCGGCAT-GGCGCAAGCCTTCGTCGGATCCCTCCATCCCAGGGTGCCCCGATACGGGTGGGAGCGGGCCTCCGGGCGTCGTTCTACCCAGCGGGCATACCCCGAGTGTGCAGGATGCGACCCGAAAGATGGTGAACTATGCCTGATCAGGTTGAAGTCAGGGGAAACCCTGATGGAGGACCGAAGCAATTCTGACGTGCAAATCGATTG

>LK-B12

TGGGACCGGTGCCACCTTCGGGTGGTGCCGCTCAATCGAACACCCATAGGCGAAGACAACTCGAAGACGCATGTCACGGGATTACGGGTTCGGCATTGGCGCAAGCCTACGTCGGATCCCTCCATCCCAGGGTGCCCCGATACGGGTGGGAGCGGGCCTCCGGGCGTCGTTCTACCCAGCGGGCATACCCCGAGTGTGCAGGATGCGACCCGAAAGATGGTGAACTATGCCTGATCAGGTTGAAGTCAGGGGAAACCCTGATGGAGGACCGAAGCAATTCTGACGTGCAAATCGATTG

>LK-B13

TGGGACCGATACCACCGGTGTGTGGTGTCGCTCAATCGAACACCCATAGGCGAAGACAACTCGATGACGTTCGTCACGGGATTACGGGTTCGGCAT-GGCGCAAGCCTTCGTCGGATCCCTCCATCCCAGGGTGCCCCGATACGGGTGGGAGCGGGCCTCCGGGCGTCGTTCTACCCAGCGGGCATACCCCGAGTGTGCAGGATGCGACCCGAAAGATGGTGAACTATGCCTGATCAGGTTGAAGTCAGGGGAAACCCTGATGGAGGACCGAAGCAATTCTGACGTGCAAATCGATTG

>LK-B14

TGGGACCGATACCACCGGTGTGTGGTGTCGCTCAATCGAACACCCATAGGCGAAGACAACTCGATGACGTTCGTCACGGGATTACGGGTTCGGCAT-GGCGCAAGCCTTCGTCGGATCCCTCCATCCCAGGGTGCCCCGATACGGGTGGGAGCGGGCCTCCGGGCGTCGTTCTACCCAGCGGGCATACCCCGAGTGTGCAGGATGCGACCCGAAAGATGGTGAACTATGCCTGATCAGGTTGAAGTCAGGGGAAACCCTGATGGAGGACCGAAGCAATTCTGACGTGCAAATCGATTG

>LK-B15

TGGGACCGATACCACCGGTGTGTGGTGTCGCTCAATCGAACACCCATAGGCGAAGACAACTCGATGACGTTCGTCACGGGATTACGGGTTCGGCAT-GGCGCAAGCCTTCGTCGGATCCCTCCATCCCAGGGTGCCCCGATACGGGTGGGAGCGGGCCTCCGGGCGTCGTTCTACCCAGCGGGCATACCCCGAGTGTGCAGGATGCGACCCGAAAGATGGTGAACTATGCCTGATCAGGTTGAAGTCAGGGGAAACCCTGATGGAGGACCGAAGCAATTCTGACGTGCAAATCGATTG

>LK-B16

TGGGACCGATACCACCGGTGTGTGGTGTCGCTCAATCGAACACCCATAGGCGAAGACAACTCGATGACGTTCGTCACGGGATTACGGGTTCGGCAT-GGCGCAAGCCTTCGTCGGATCCCTCCATCCCAGGGTGCCCCGATACGGGTGGGAGCGGGCCTCCGGGCGTCGTTCTACCCAGCGGGCATACCCCGAGTGTGCAGGATGCGACCCGAAAGATGGTGAACTATGCCTGATCAGGTTGAAGTCAGGGGAAACCCTGATGGAGGACCGAAGCAATTCTGACGTGCAAATCGATTG

>LK-B17

TGGGACCGATACCACCGGTGTGTGGTGTCGCTCAATCGAACACCCATAGGCGAAGACAACTCGATGACGTTCGTCACGGGATTACGGGTTCGGCAT-GGCGCAAGCCTTCGTCGGATCCCTCCATCCCAGGGTGCCCCGATACGGGTGGGAGCGGGCCTCCGGGCGTCGTTCTACCCAGCGGGCATACCCCGAGTGTGCAGGATGCGACCCGAAAGATGGTGAACTATGCCTGATCAGGTTGAAGTCAGGGGAAACCCTGATGGAGGACCGAAGCAATTCTGACGTGCAAATCGATTG

>LK-B18

TGGGACCGATACCACCGGTGTGTGGTGTCGCTCAATCGAACACCCATAGGCGAAGACAACTCGATGACGTTCGTCACGGGATTACGGGTTCGGCAT-GGCGCAAGCCTTCGTCGGATCCCTCCATCCCAGGGTGCCCCGATACGGGTGGGAGCGGGCCTCCGGGCGTCGTTCTACCCAGCGGGCATACCCCGAGTGTGCAGGATGCGACCCGAAAGATGGTGAACTATGCCTGATCAGGTTGAAGTCAGGGGAAACCCTGATGGAGGACCGAAGCAATTCTGACGTGCAAATCGATTG

>LK-B19

TGGGACCGATACCACCGGTGTGTGGTGTCGCTCAATCGAACACCCATAGGCGAAGACAACTCGATGACGTTCGTCACGGGATTACGGGTTCGGCAT-GGCGCAAGCCTTCGTCGGATCCCTCCATCCCAGGGTGCCCCGATACGGGTGGGAGCGGGCCTCCGGGCGTCGTTCTACCCAGCGGGCATACCCCGAGTGTGCAGGATGCGACCCGAAAGATGGTGAACTATGCCTGATCAGGTTGAAGTCAGGGGAAACCCTGATGGAGGACCGAAGCAATTCTGACGTGCAAATCGATTG

>LK-B20

TGGGACCGATACCACCGGTGTGTGGTGTCGCTCAATCGAACACCCATAGGCGAAGACAACTCGATGACGTTCGTCACGGGATTACGGGTTCGGCAT-GGCGCAAGCCTTCGTCGGATCCCTCCATCCCAGGGTGCCCCGATACGGGTGGGAGCGGGCCTCCGGGCGTCGTTCTACCCAGCGGGCATACCCCGAGTGTGCAGGATGCGACCCGAAAGATGGTGAACTATGCCTGATCAGGTTGAAGTCAGGGGAAACCCTGATGGAGGACCGAAGCAATTCTGACGTGCAAATCGATTG

>LK-B21

TGGGACCGATACCACCGGTGTGTGGTGTCGCTCAATCGAACACCCATAGGCGAAGACAACTCGATGACGTTCGTCACGGGATTACGGGTTCGGCAT-GGCGCAAGCCTTCGTCGGATCCCTCCATCCCAGGGTGCCCCGATACGGGTGGGAGCGGGCCTCCGGGCGTCGTTCTACCCAGCGGGCATACCCCGAGTGTGCAGGATGCGACCCGAAAGATGGTGAACTATGCCTGATCAGGTTGAAGTCAGGGGAAACCCTGATGGAGGACCGAAGCAATTCTGACGTGCAAATCGATTG

>LK-B22

TGGGACCGATACCACCGGTGTGTGGTGTCGCTCAATCGAACACCCATAGGCGAAGACAACTCGATGACGTTCGTCACGGGATTACGGGTTCGGCAT-GGCGCAAGCCTTCGTCGGATCCCTCCATCCCAGGGTGCCCCGATACGGGTGGGAGCGGGCCTCCGGGCGTCGTTCTACCCAGCGGGCATACCCCGAGTGTGCAGGATGCGACCCGAAAGATGGTGAACTATGCCTGATCAGGTTGAAGTCAGGGGAAACCCTGATGGAGGACCGAAGCAATTCTGACGTGCAAATCGATTG

>LK-B23

TGGGACCGATACCACCGGTGTGTGGTGTCGCTCAATCGAACACCCATAGGCGAAGACAACTCGATGACGTTCGTCACGGGATTACGGGTTCGGCAT-GGCGCAAGCCTTCGTCGGATCCCTCCATCCCAGGGTGCCCCGATACGGGTGGGAGCGGGCCTCCGGGCGTCGTTCTACCCAGCGGGCATACCCCGAGTGTGCAGGATGCGACCCGAAAGATGGTGAACTATGCCTGATCAGGTTGAAGTCAGGGGAAACCCTGATGGAGGACCGAAGCAATTCTGACGTGCAAATCGATTG

>LK-B24

TGGGACCGATACCACCGGTGTGTGGTGTCGCTCAATCGAACACCCATAGGCGAAGACAACTCGATGACGTTCGTCACGGGATTACGGGTTCGGCAT-GGCGCAAGCCTTCGTCGGATCCCTCCATCCCAGGGTGCCCCGATACGGGTGGGAGCGGGCCTCCGGGCGTCGTTCTACCCAGCGGGCATACCCCGAGTGTGCAGGATGCGACCCGAAAGATGGTGAACTATGCCTGATCAGGTTGAAGTCAGGGGAAACCCTGATGGAGGACCGAAGCAATTCTGACGTGCAAATCGATTG

>LK-B25

TGGGACCGATACCACCGGTGTGTGGTGTCGCTCAATCGAACACCCATAGGCGAAGACAACTCGATGACGTTCGTCACGGGATTACGGGTTCGGCAT-GGCGCAAGCCTTCGTCGGATCCCTCCATCCCAGGGTGCCCCGATACGGGTGGGAGCGGGCCTCCGGGCGTCGTTCTACCCAGCGGGCATACCCCGAGTGTGCAGGATGCGACCCGAAAGATGGTGAACTATGCCTGATCAGGTTGAAGTCAGGGGAAACCCTGATGGAGGACCGAAGCAATTCTGACGTGCAAATCGATTG

>LK-B26

TGGGACCGATACCACCGGTGTGTGGTGTCGCTCAATCGAACACCCATAGGCGAAGACAACTCGATGACGTTCGTCACGGGATTACGGGTTCGGCAT-GGCGCAAGCCTTCGTCGGATCCCTCCATCCCAGGGTGCCCCGATACGGGTGGGAGCGGGCCTCCGGGCGTCGTTCTACCCAGCGGGCATACCCCGAGTGTGCAGGATGCGACCCGAAAGATGGTGAACTATGCCTGATCAGGTTGAAGTCAGGGGAAACCCTGATGGAGGACCGAAGCAATTCTGACGTGCAAATCGATTG

>LK-B27

TGGGACCGGTGCCACCTTCGGGTGGTGCCGCTCAATCGAACACCCATAGGCGAAGACAACTCGAAGACGCATGTCACGGGATTACGGGTTCGGCATTGGCGCAAGCCTACGTCGGATCCCTCCATCCCAGGGTGCCCCGATACGGGTGGGAGCGGGCCTCCGGGCGTCGTTCTACCCAGCGGGCATACCCCGAGTGTGCAGGATGCGACCCGAAAGATGGTGAACTATGCCTGATCAGGTTGAAGTCAGGGGAAACCCTGATGGAGGACCGAAGCAATTCTGACGTGCAAATCGATTG

>LK-B28

TGGGACCGATACCACCGGTGTGTGGTGTCGCTCAATCGAACACCCATAGGCGAAGACAACTCGATGACGTTCGTCACGGGATTACGGGTTCGGCAT-GGCGCAAGCCTTCGTCGGATCCCTCCATCCCAGGGTGCCCCGATACGGGTGGGAGCGGGCCTCCGGGCGTCGTTCTACCCAGCGGGCATACCCCGAGTGTGCAGGATGCGACCCGAAAGATGGTGAACTATGCCTGATCAGGTTGAAGTCAGGGGAAACCCTGATGGAGGACCGAAGCAATTCTGACGTGCAAATCGATTG

>LK-B29

TGGGACCGGTGCCACCTTCGGGTGGTGCCGCTCAATCGAACACCCATAGGCGAAGACAACTCGAAGACGCATGTCACGGGATTACGGGTTCGGCATTGGCGCAAGCCTACGTCGGATCCCTCCATCCCAGGGTGCCCCGATACGGGTGGGAGCGGGCCTCCGGGCGTCGTTCTACCCAGCGGGCATACCCCGAGTGTGCAGGATGCGACCCGAAAGATGGTGAACTATGCCTGATCAGGTTGAAGTCAGGGGAAACCCTGATGGAGGACCGAAGCAATTCTGACGTGCAAATCGATTG

>LK-B30

TGGGACCGGTGCCACCTTCGGGTGGTGCCGCTCAATCGAACACCCATAGGCGAAGACAACTCGAAGACGCATGTCACGGGATTACGGGTTCGGCATTGGCGCAAGCCTACGTCGGATCCCTCCATCCCAGGGTGCCCCGATACGGGTGGGAGCGGGCCTCCGGGCGTCGTTCTACCCAGCGGGCATACCCCGAGTGTGCAGGATGCGACCCGAAAGATGGTGAACTATGCCTGATCAGGTTGAAGTCAGGGGAAACCCTGATGGAGGACCGAAGCAATTCTGACGTGCAAATCGATTG

>LK-C1

TGGGACCGGTGCCACCTTCGGGTGGTGCCGCTCAATCGAACACCCATAGGCGAAGACAACTCGAAGACGCATGTCACGGGATTACGGGTTCGGCATTGGCGCAAGCCTACGTCGGATCCCTCCATCCCAGGGTGCCCCGATACGGGTGGGAGCGGGCCTCCGGGCGTCGTTCTACCCAGCGGGCATACCCCGAGTGTGCAGGATGCGACCCGAAAGATGGTGAACTATGCCTGATCAGGTTGAAGTCAGGGGAAACCCTGATGGAGGACCGAAGCAATTCTGACGTGCAAATCGATTG

>LK-C2

TGGGACCGGTGCCACCTTCGGGTGGTGCCGCTCAATCGAACACCCATAGGCGAAGACAACTCGAAGACGCATGTCACGGGATTACGGGTTCGGCATTGGCGCAAGCCTACGTCGGATCCCTCCATCCCAGGGTGCCCCGATACGGGTGGGAGCGGGCCTCCGGGCGTCGTTCTACCCAGCGGGCATACCCCGAGTGTGCAGGATGCGACCCGAAAGATGGTGAACTATGCCTGATCAGGTTGAAGTCAGGGGAAACCCTGATGGAGGACCGAAGCAATTCTGACGTGCAAATCGATTG

>LK-C3

TGGGACCGGTGCCACCTTCGGGTGGTGCCGCTCAATCGAACACCCATAGGCGAAGACAACTCGAAGACGCATGTCACGGGATTACGGGTTCGGCATTGGCGCAAGCCTACGTCGGATCCCTCCATCCCAGGGTGCCCCGATACGGGTGGGAGCGGGCCTCCGGGCGTCGTTCTACCCAGCGGGCATACCCCGAGTGTGCAGGATGCGACCCGAAAGATGGTGAACTATGCCTGATCAGGTTGAAGTCAGGGGAAACCCTGATGGAGGACCGAAGCAATTCTGACGTGCAAATCGATTG

>LK-C4

TGGGACCGGTGCCACCTTCGGGTGGTGCCGCTCAATCGAACACCCATAGGCGAAGACAACTCGAAGACGCATGTCACGGGATTACGGGTTCGGCATTGGCGCAAGCCTACGTCGGATCCCTCCATCCCAGGGTGCCCCGATACGGGTGGGAGCGGGCCTCCGGGCGTCGTTCTACCCAGCGGGCATACCCCGAGTGTGCAGGATGCGACCCGAAAGATGGTGAACTATGCCTGATCAGGTTGAAGTCAGGGGAAACCCTGATGGAGGACCGAAGCAATTCTGACGTGCAAATCGATTG

>LK-C5

TGGGACCGGTGCCACCTTCGGGTGGTGCCGCTCAATCGAACACCCATAGGCGAAGACAACTCGAAGACGCATGTCACGGGATTACGGGTTCGGCATTGGCGCAAGCCTACGTCGGATCCCTCCATCCCAGGGTGCCCCGATACGGGTGGGAGCGGGCCTCCGGGCGTCGTTCTACCCAGCGGGCATACCCCGAGTGTGCAGGATGCGACCCGAAAGATGGTGAACTATGCCTGATCAGGTTGAAGTCAGGGGAAACCCTGATGGAGGACCGAAGCAATTCTGACGTGCAAATCGATTG

>LK-D1

TGGGACCGGTGCCACCTTCGGGTGGTGCCGCTCAATCGAACACCCATAGGCGAAGACAACTCGAAGACGCATGTCACGGGATTACGGGTTCGGCATTGGCGCAAGCCTACGTCGGATCCCTCCATCCCAGGGTGCCCCGATACGGGTGGGAGCGGGCCTCCGGGCGTCGTTCTACCCAGCGGGCATACCCCGAGTGTGCAGGATGCGACCCGAAAGATGGTGAACTATGCCTGATCAGGTTGAAGTCAGGGGAAACCCTGATGGAGGACCGAAGCAATTCTGACGTGCAAATCGATTG

>LK-D2

TGGGACCGGTGCCACCTTCGGGTGGTGCCGCTCAATCGAACACCCATAGGCGAAGACAACTCGAAGACGCATGTCACGGGATTACGGGTTCGGCATTGGCGCAAGCCTACGTCGGATCCCTCCATCCCAGGGTGCCCCGATACGGGTGGGAGCGGGCCTCCGGGCGTCGTTCTACCCAGCGGGCATACCCCGAGTGTGCAGGATGCGACCCGAAAGATGGTGAACTATGCCTGATCAGGTTGAAGTCAGGGGAAACCCTGATGGAGGACCGAAGCAATTCTGACGTGCAAATCGATTG

>LK-D4

TGGGACCGGTGCCACCTTCGGGTGGTGCCGCTCAATCGAACACCCATAGGCGAAGACAACTCGAAGACGCATGTCACGGGATTACGGGTTCGGCATTGGCGCAAGCCTACGTCGGATCCCTCCATCCCAGGGTGCCCCGATACGGGTGGGAGCGGGCCTCCGGGCGTCGTTCTACCCAGCGGGCATACCCCGAGTGTGCAGGATGCGACCCGAAAGATGGTGAACTATGCCTGATCAGGTTGAAGTCAGGGGAAACCCTGATGGAGGACCGAAGCAATTCTGACGTGCAAATCGATTG

>LK-D5

TGGGACCGGTGCCACCTTCGGGTGGTGCCGCTCAATCGAACACCCATAGGCGAAGACAACTCGAAGACGCATGTCACGGGATTACGGGTTCGGCATTGGCGCAAGCCTACGTCGGATCCCTCCATCCCAGGGTGCCCCGATACGGGTGGGAGCGGGCCTCCGGGCGTCGTTCTACCCAGCGGGCATACCCCGAGTGTGCAGGATGCGACCCGAAAGATGGTGAACTATGCCTGATCAGGTTGAAGTCAGGGGAAACCCTGATGGAGGACCGAAGCAATTCTGACGTGCAAATCGATTG

>AY601516 Sund Andaman IN

TGGGACCGATACCACCGGTGTGTGGTGTCGCTCAATCGAACACCCATAGGCGAAGACAACTCGATGACGTTCGTCACGGGATTACGGGTTCGGCAT-GGCGCAAGCCTTCGTCGGATCCCTCCATCCCAGGGTGCCCCGATACGGGTGGGAGCGGGCCTCCG

GGCGTCGTTCTACCCAGCGGGCATACCCCGAGTGTGCAGGATGCGACCCGAAAGATGGTGAACTATGCCTGATCAGGTTGAAGTCAGGGGAAACCCTGATGGAGGACCGAAGCAATTCTGACGTGCAAATCGATTG

>AY691512 Sund Nicobar1 IN

TGGGACCGATACCACCGGTGTGTGGTGTCGCTCAATCGAACACCCATAGGCGAAGACAACTCGATGACGTTCGTCACGGGATTACGGGTTCGGCAT-GGCGCAAGCCTTCGTCGGATCCCTCCATCCCAGGGTGCCCCGATACGGGTGGGAGCGGGCCTCCG

GGCGTCGTTCTACCCAGCGGGCATACCCCGAGTGTGCAGGATGCGACCCGAAAGATGGTGAACTATGCCTGATCAGGTTGAAGTCAGGGGAAACCCTGATGGAGGACCGAAGCAATTCTGACGTGCAAATCGATTG

>AY691513 Sund Nicobar2 IN

TGGGACCGATACCACCGGTGTGTGGTGTCGCTCAATCGAACACCCATAGGCGAAGACAACTCGATGACGTTCGTCACGGGATTACGGGTTCGGCAT-GGCGCAAGCCTTCGTCGGATCCCTCCATCCCAGGGTGCCCCGATACGGGTGGGAGCGGGCCTCCG

GGCGTCGTTCTACCCAGCGGGCATACCCCGAGTGTGCAGGATGCGACCCGAAAGATGGTGAACTATGCCTGATCAGGTTGAAGTCAGGGGAAACCCTGATGGAGGACCGAAGCAATTCTGACGTGCAAATCGATTG

>FJ457630.1 Vagus China

TGGGACCGGTGCCACCTCCGGGTGGTGCCGCTCAATCGAACACCCATAGGCGAAGACAACTCGAAGACGTATGTCACGGGATTACGGGTTCGGCATTGGCGCAAGCCTTCGTCGGATCCCTCCATCCCAGGGTGCCCCGATACGGGTGGGAGCGGGCCTCCGGGCGTCGTTCTACCCAGCGGGCATACCCCGAGTGTGCAGGATGCGACCCGAAAGATGGTGAACTATGCCTGATCAGGTTGAAGTCAGGGGAAACCCTGATGGAGGACCGAAGCAATTCTGACGTGCAAATCGATTG
