# Supplementary material for: Genetic evidence for malaria vectors of the Anopheles sundaicus complex in Sri Lanka with morphological characteristics attributed to Anopheles subpictus species B
Source: Malar J. 2010 Nov 29;9:343. doi: 10.1186/1475-2875-9-343 (PMC3009661; doi:10.1186/1475-2875-9-343)
Supplement: Additional File 3 — ITS-2 sequences used for phylogenetic analysis. Sequences of the ITS-2 region of rDNA used for phylogenetic analysis. LK- B series refer to individual specimens collected in the study that were initially morphologically characterized as belonging to An. subpictus species B. LK-A series refers to those similarly characterized as An, subpictus species A. Other sequences of An. sundaicus s.l. (Sund), An subpictus (Subp) and An. vagus (Vagus) from different locations in countries indicated by their standard two letter abbreviations, were obtained from GenBank. [file 1475-2875-9-343-S3.DOC]

**ITS-2 sequences used for phylogenetic analysis**

>GQ870337.1 Subp Inland LK

CCTACTAGGTACTGAGAGATTCCTATAACTTGACTACAGACGGCGCCACA

AACGGGCTGACGGGCCATCCGTCGTCCGGCGTGCGACTGTGCAGCATGGCGTGCTCGGGTCTCGGCGTGG

ACCCTTGGGCGCTGAAAGTGGACACTGTTTGGCGGCACCTGCGCGTGTGCTCTCAGTGTTGACGTATGGT

GAGGGTAGTGTCAAATCGCACGGTTCGACAACAAGCGTACCGTCGAGTTTGGTGCAATCGGATGCCTACT

ACCATGGGCGGAGCCGGCGTGCATTCAACACTCGACGTCCTGTATCAACCGGATGCCAACTTGGTTGGTG

GTGCCGGCGCAGACAGGACACTGAATCGATCTTGGTGGTACAACCCACATGTGGGTTAGTAGGTAGGTGG

TCGATGTGTGCAGGTGACAACCGGATGCCAGCGATGGCGGTGCCGGCGCACACCAGCACACTCGCCCCTA

GGTCGCTTGTTGCGTGTAACGCGTGTGATCCATACACATACCTGTTTGAGCTGTGCGTTGAACACAAGAG

GATGAGAGTTGCCAAACACACAGACCACACTCCAGT

>GQ870336.1 Subp Inland LK

CCTACTAGGTACTGAGAGATTCCTATAACTTGACTACAGACGGCGCCACA

AACGGGCTGACGGGCCATCCGTCGTCCGGCGTGCGACTGTGCAGCATGGCGTGCTCGGGTCTCGGCGTGG

ACCCTTGGGCGCTGAAAGTGGACACTGTTTGGCGGCACCTGCGCGTGTGCTCTCAGTGTTGACGTATGGT

GAGGGTAGTGTCAAATCGCACGGTTCGACAACAAGCGTACCGTCGAGTTTGGTGCAATCGGATGCCTACT

ACCATGGGCGGAGCCGGCGTGCATTCAACACTCGACGTCCTGTATCAACCGGATGCCAACTTGGTTGGTG

GTGCCGGCGCAGACAGGACACTGAATCGATCTTGGTGGTACAACCCACATGTAGGTGGTCGATGTGTGCA

GGTGACAACCGGATGCCAGCGATGGCGGTGCCGGCGCACACCAGCACACTCGCCCCTAGGTCGCTTGTTG

CGTGTAACGCGTGTGATCCATACACATACCTGTTTGAGCTGTGCGTTGAACACAAGAGGATGAGAGTTGC

CAAACACACAGACCACACTCCAGT

>GQ870335.1 Subp Java ID

CCTACTAGGTACTTCGATTTTCCTATAATTAGACTACAGACGGGCGCCAC

TAACGGGCTGACGGGTTATCCGTCGTCTGGCGTGCGACTGTGCAGCATGGCGTGCTCGGGTGCTCAGCGT

GCACCCTTGGGCGCTGAAAGTGTGGATACTTTGTTTGAGCGGCACCTTTGCGTGTGCTCTCTCCTAAGTG

TCGACGTATGGTGAGGGTAGTGTCAAGCCGCACGGTGCGACAACACAAGCGTACTGTCGAGTTTGGTGCA

ATCGGATGCCTACTACCATGGGCGGTGCCGGCGTGCATTCAACACTCGACGACGTGCGTGCGTGCGTGTG

TCCTGTATCAACCGGATGCCAACTGCTGTCAGTTGGTGGTGTCGGCGCAGACAGGACGCTCGCGCGCGTA

CGCTTGAGTCGTGTAACGCGTGCGACCCATACACTTACCTGCTTGAGCTGTGCGTTGCGAGCTGGAGAGA

GTTGCCAGACGGAGCAACAAATACCACCTACTTTCCAGT

>GQ870334.1 Subp Myanmar

CCTACTAGGTACTTCGATTTTCCTATAATTAGACTACAGACGGGCGCCAC

TAATGGGCTGACGGGTTATCCGTCGTCTGGCGTGCGACTGTGCAGCATGGCGTGCTCGGGTCTCGGCGTG

GACCCTTGGGCGCTGAAAGTGGATACTCTGTTTGAGCGGCACCTTTGCGTGTGCTCTCCTAAGTGTCGAC

GTATGGTGAGGGTAGTGTCAAGCCGCACGGTGCGACAACACAAGCGTACTGTCGAGTTTGGTGCAATCGG

ATGCCTACTACCATGGGCGGTGCCGGCGTGCATTCAACACTCGACGTGTGCGTCCTGTATCAACCGGATG

CCAACTGCTGTCAGTTGGTGGTGTCGGCGCAGACAGGACGCGCGCGTACGCTTGAGTCGTGTAACGCGTG

CGACCCATACACGTACCTGCTTGAGCTATGCGTTGCGAGTTGGAGAGTTGCCAGACGGAGGAAATACCAC

AACTACTCCAGT

>GQ870333.1 Subp Thailand

CCTACTAGGTACTTCGATTTTCCTATACTTAGACTACAGACGGGCGCCAC

TAACGGGCTGACGGGTTATCCGTCGTCTGGCGTGCGACTGTGCAGCATGGCGTGCTCGGGTCTCGGCGTG

GACCCTTGGGCGCTGAAAGTGGATACTCTGTTTGAGCGGCACCTTTGCGTGTGCTCTCCTAAGTGTCGAC

GTATGGTGAGGGTAGTGTCAAGCCGCACGGTGCGACAACACAAGCGTACTGTCGAGTTTGGTGCAATCGG

ATGCCTACTACCATGGGCGGTGCCGGCGTGCATTAAACACTCGACGTGCGTGTCCTGTATCAACCGGATG

CCAACTGCTGTCAGTTGGTGGTGTCGGCGCAGACAGGACGCGCGCGTACGCTTGAGTCGTGTAACGCGTG

CGACCCATACACGTACCTGCTTGAGCTATGCGTTGCGAGTTGGAGAGTTGCCAGACGGAGGAGATACCAC

AAACTCCAGT

>GQ870330.1 Subp Vietnam

CCTACTAGGTACTTCGATTTTCCTATAATTAGACTACAGACGGGCGCCAC

TAACGGGCTGACGGGTTATCCGTCGTCTGGCGTGCGACTGTGCAGCATGGCGTGCTCGGGTCTCGGCGTG

GACCCTTGGGCGCTGAAAGTGGATACTCTGTTTGAGCGGCACCTTTGCGTGTGCTCTCCTAAGTGTCGAC

GTATGGTGAGGGTAGTGTCAAGCCGCACGGTGCGACAACACAAGCGTACTGTCGAGTTTGGTGCAATCGG

ATGCCTACTACCATGGGCGGTGCCGGCGTGCATTCAACACTCGACGTGTGTGTCCTGTATCAACCGGATG

CCAACTGCTGTCAGTTGGTGGTGTCGGCGCAGACAGGACGCGCGCGTACGCTTGAGTCGTGTAACGCGTG

CGACCCATACACGTACCTGCTTGAGCTATGCGTTGCGAGTTGGAGAGTTGCCAGACGGAGCAAATACCAC

ACCTACTCCAGT

>GQ870329.1 Subp Cambodia

CCTACTAGGTACTTCGATTTTCCTATAATTAGACTACAGACGGGCGCCAC

TAACGGGCTGACGGGTTATCCGTCGTCTGGCGTGCGACTGTGCAGCATGGCGTGCTCGGGTCTCGGCGTG

GACCCTTGGGCGCTGAAAGTGGATACTCTGTTTGAGCGGCACCTTTGCGTGTGCTCTCCTAAGTGTCGAC

GTATGGTGAGGGTAGTGTCAAGCCGCACGGTGCGACAACACAAGCGTACTGTCGAGTTTGGTGCAATCGG

ATGCCTACTACCATGGGCGGTGCCGGCGTGCATTCAACACTCGACGTGTGTGTCCTGTATCAACCGGATG

CCRACTGCTGTCAGTTGGTGGTGTCGGCGCAGACAGGACGCGCGCGTACGCTTGAGTCGTGTAACGCGTG

CGACCCATACACGTACCTGCTTGAGCTATGCGTTGCGAGTTGGAGAGTTGCCAGACGGAGCAAATACCAC

ACCTACTCCAGT

>GQ870328.1 Subp Flores ID

CCTACTAGGTACTTCGATTTTCCTATACTTAGACTACAGACGGGCGCCAC

TAACGGGCTGACGGGTTATCCGTCGTCTGGCGTGCGACTGTGCAGCATGGCGTGCTCGGGTCTCGGCGTG

GACCCTTGGGCGCTGAAAGTGGATACTCTGTTTGAGCGGCACCTTTGCGTGTGCTCTCCTAAGTGTCGAC

GTATGGTGAGGGTAGTGTCAAGCCGCACGGTGCGACAACACAAGCGTACTGTCGAGTTTGGTGCAATCGG

ATGCCTACTACCATGGGCGGTGCCGGCGTGCATTAAACACTCGACGTGCGTGTCCTGTATCAACCGGATG

CCAACTGCTGTGTCAGTTGGTGGTGTCGGCGCAGACAGGACGCGCGCGTACGCTTGAGTCGTGTAACGCG

TGCGACCCATACACGTACCTGCTTGAGCTATGCGTTGCGAGTTGGAGAGTTGCCAGACGGAGGAGATACC

ACAAACACTCCAGT

>GQ870326.1 Subp Cambodia

CCTACTAGGTACTTCGATTTTCCTATACTTAGACTACAGACGGGCGCCAC

TAACGGGCTGACGGGTTATCCGTCGTCTGGCGTGCGACTGTGCAGCATGGCGTGCTCGGGTCTCGGCGTG

GACCCTTGGGCGCTGAAAGTGGATACTCTGTTTGAGCGGCACCTTTGCGTGTGCTCTCCTAAGTGTCGAC

GTATGGTGAGGGTAGTGTCAAGCCGCACGGTGCGACAACACAAGCGTACTGTCGAGTTTGGTGCAATCGG

ATGCCTACTACCATGGGCGGTGCCGGCGTGCATTAAACACTCGACGTGCGTGTCCTGTATCAACCGGATG

CCAACTGCTGTCAGTTGGTGGTGTCGGCGCAGACAGGACGCGCGCGTACGCTTGAGTCGTGTAACGCGTG

CGACCCATACACGTACCTGCTTGAGCTATGCGTTGCGAGTTGGAGAGTTGCCAGACGGAGGAGATACCAC

AAACTCTCCAGT

>GQ870325.1 Subp Vietnam

CCTACTARGTACTTCGATTTTCCTATACTTAGACTACAGACGGGCGCCAC

TAACGGGCTGACGGGTTATCCGTCGTCTGGCGTGCGACTGTGCAGCATGGCGTGCTCGGGTCTCGGCGTG

GACCCTTGGGCGCTGAAAGTGGATACTCTGTTTGAGCGGCACCTTTGCGTGTGCTCTCCTAAGTGTCGAC

GTATGGTGAGGGTAGTGTCAAGCCGCACGGTGCGACAACACAAGCGTACTGTCGAGTTTGGTGCAATCGG

ATGCCTACTACCATGGGCGGTGCCGGCGTGCATTAAACACTCGACGTGCGTGTCCTGTATCAACCGGATG

CCAACTGCTGTCAGTTGGTGGTGTCGGCGCAGACAGGACGCGCGCGTACGCTTGAGTCGTGTAACGCGTG

CGACCCATACACGTACCTGCTTGAGCTATGCGTTGCGAGTTGGAGAGTTGCCAGACGGAGGAGATACCAC

AAACTCTCCAGT

>EF601870.1 Subp Punjab1 IN

CCTACTAAGTTCTGAGAGATTCCTATAACTTGACTACCCACGGCGCCTCAAACAGGCTGACGGGCCATCC

GTCGTCCGGCGTGCGACTGTGCAGCATGGCGTGCTCGGGTCTCGGCGTGGACCCTTGGGCGCTGAAAGTGGACACTGTTTGGCGGCACCTGCGCGTGTGCTCTCAGTGTTGACGTATGGTGAGGGTAGTGTCAAATCGCACGGTTCGACAACAAGCGTACCGTCGAGTTTGGTGCAATCGGATGCCTACTACCATGGGCGGAGCCGGCGTGCATTCAACACTCGACGTCCTGTATCAACCGGATGCCAACTTGGTTGGTGGTGCCGGCGCAGACAGGACACTGAATCGA

TCTTGGTGGTACAACCCACATGTGGGTGGTCAATGTGTGCAGGTGACAACCGGATGCCAGCGATGGCGGT

GCCGGCGCACACCAGCACACTCGCCCCTAGGTCGCTTGTTGCGTGTAACGCGTGTGATCCATACACATAC

CTGTTTGAGCTGTGCGTTGAACACAAGAGGATGAGAGTTGCCAAACACACAGACCACACTCCAGT

>EF601869.1 Subp Punjab2 IN

CCTACTAGGTTCTGAGAGATTCCTATAACTTGACTACCGACGGCGCCACAAACGGGCTGACGGGCCATCCGTCGTCCGGCGTGCGACTGTGCAGCATGGCGTGCTCGGGTCTCGGCGTGGACCCTTGGGCGCTGAAAGTGGACACTGTTTGGCGGCACCTGCGCGTGTGCTCTCAGTGTTGACGTATGGTGAGGGTAGTGTCAAATCGCACGGTTCGACAACAAGCGTACCGTCGAGTTTGGTGCAATCGGATGCCTACTACCATGGGCGGAGCCGGCGTGCATTCAACA

CTCGACGTCCTGTATCAACCGGATGCCAACTTGGTTGGTGGTGCCGGCGCAGACAGGACACTGAATCGAT

CTTGGTGGTACAACCCACATGTGGGTTAGTAGGTAGGTGGTCGATGTGTGCAAGTCACACCAGGATGCCG

CCGGTGCACACCACCGCACTCACCCCTAGATTCCTCGTTGCGTGTATGGGGGGTGATCCATACACTGATC

TATATACACTCTGCTTTGAACTCAAGATGAAAAGAGTTGCCAAAAACACTTGCCCACACTCCAGA

>EF601868.1 Subp Punjab3 IN

CCTACTAGGTACTGAGAGATTCCTATAACTTGACTACAGACGGCGCCACAAACGGGCTGACGGGCCATCCGTCGTCCGGCGTGCGACTGTGCAGCATGGCGTGCTCGGGTCTCGGCGTGGACCCTTGGGCGCTGAAAGTGGACACTGTTTGGCGGCACCTGCGCGTGTGCTCTCAGTGTTGACGTATGGTGAGGGTAGTGTCAAATCGCACGGTTCG

ACAACAAGCGTACCGTCGAGTTTGGTGCAATCGGATGCCTACTACCATGGGCGGAGCCGGCGTGCATTCA

ACACTCGACGTCCTGTATCAACCGGATGCCAACTTGGTTGGTGGTGCCGGCGCAGACAGGACACTGAATC

GATCTTGGTGGTACAACCCACATGTGGGTGATTAAGTAGGTGGTGTAAGTACCGGATGCCAACCATGGCG

GTGCCGGCGCACACCACCACACTCACCCCTAGGTCGCTTGTTGCGTGTAACGCGTGTGATCCATACACAT

ACCTGTTTGAGCTGTGCGTTGAACACAAGAGGATGAGAGTTGCCAAACACACAGACCACACTCCAGT

>AY049004.1 Subp Coast LK

CCTACTAGGTACTTCGATTTTCCTATAATTAGACTACAGACGGGCGCCACTAATGGGCTGACGGGTTATCCGTCGTCTGGCGTGNGACTGTGCAGCATGGCGTGCTCGGGTCTCGGCGTGGACCCTTGGGCGCTGAAAGTGGATACTCTGTTTGAGCGGCACCTTTGCGTGTGCTCTCCTAAGTGTCGACGTATGGTGAGGGTATTGTCAAGCCGCACGGTGCGACAACACAAGCGTACTGTCGAGTTTGGTGCAATCGGATGCCTACTACCATGGGCGGTGCCGGCGTGCATTCAACAATCGACGTGTGCGTCCTGTATCAACCGGATGCCAACTGCTGTCAGTTGGTGGTGTCCGCGCAAACAGGACGCGCGCGTACGCTTGAGTCGTGTAACGCGTGCGACCCATACACGTACCTGCTTGAACTATGCGTTGCGAATTGGAAAGTTGCCAGAAGGAAGGAAATACTACAATTACTGGCCGTAGGCCTTCAAGTGAATGTTGACTACCCCCTAAATTTAAGCAT

>AF406615.1 Subp Inland LK

CCTACTAGGTACTTCGATTTTCCTATAATTAGACTACAGACGGGCGCCACTAATGGGCTGACGGGTTA

TCCGTCGTCTGGCGTGCGACTGTGCAGCATGGCGTGCTCGGGTCTCGGCGTGGACCCTTGGGCGCTGAAA

GTGGATACTCTGTTTGAGCGGCACCTTTGCGTGTGCTCTCCTAAGTGTCGACGTATGGTGAGGGTAGTGT

CAAGCCGCACGGTGCGACAACACAAGCGTACTGTCGAGTTTGGTGCAATCGGAATGCCTACTACCATGGG

CGGTGCCGGCGTGCATTCAACACTCGACGTGTGCGTCCTGTATCAACCGGATGCCAACTGCTGTCAGTTG

GTGGTGTCGGCGCAGACAGGACGCGCGCGTACGCTTGAGTCGTGTAACGCGTGCGACCCATACACGTACC

TGCTTGAGCTATGCGTTGCGAGTTGGAGAGTTGCCAGACGGAGGAAATACCACAATATCCAGT

>AF406616.2 Subp Coast LK

CCTACTAGGTACTTCGATTTTCCTATAATTAGACTACAGACGGGCGCCACTAATGGGCTGACGGGTTA

TCCGTCGTCTGGCGTGNGACTGTGCAGCATGGCGTGCTCGGGTCTCGGCGTGGACCCTTGGGCGCTGAAA

GTGGATACTCTGTTTGAGCGGCACCTTTGCGTGTGCTCTCCTAAGTGTCGACGTATGGTGAGGGTATTGT

CAAGCCGCACGGTGCGACAACACAAGCGTACTGTCGAGTTTGGTGCAATCGGATGCCTACTACCATGGGC

GGTGCCGGCGTGCATTCAACAATCGACGTGTGCGTCCTGTATCAACCGGATGCCAACTGCTGTCAGTTGG

TGGTGTCCGCGCAAACAGGACGCGCGCGTACGCTTGAGTCGTGTAACGCGTGCGACCCATACACGTACCT

GCTTGAACTATGCGTTGCGAATTGGAAAGTTGCCAGAAGGAAGGAAATACTACAATTACTGGCCGTAGGC

CTTCAAGTGAATGTTGACTACCCCCTAAATTTAAGCAT

>AF406614.1 Subp Inland LK

CCTACTAGAGTACTGACAAATTCCTATAACTTGACTACAGACGGCGCCACAAACGGGCTGACGGGCCAT

CCGTCGTCCGGGGTGCGACTGTGCAGCATGGCGTGCTCGGGTCTCGGCGTGGACCCTTGGGCGCTGAAAG

TGGACACTGTTTGGCGGCACCTGCGCGTGTGCTCTCATTGTTGACGTATGGTGAGGGTATTGTCAAATCG

CACGGTTCGACAACAAGCGTACCGTCGAGTTTGGTGCAATCGGATGCCTACTACCATGGGCGGAGCCGGG

GTGCATTCAACACTCGACGTCTTGTATCAACCGGATGCCAACTTGGTTGGTGGTGCCGGCGCAAACAGGA

CACTTGAATCAACCTTGGTGGTACACCCCACATGTTGGTGGTCAAGTTGTGGTGTAACTATCGGAGTCAA

AACATGATGGCGGCGATGGACGCCACAGCACTCACCCCCTACGTCCCTCGCTGAGTGTATCGTGTGTTAT

CCATCCATACACATACCTGTTTGAGCGTTGCGTTGAACACAAGAGGATGAGAGTTGCAAAACACACAGAC

CACACTCCAGT

>AF406613.1 Subp Inland LK

CCTACTAGAGTACTGACAAATTCCTATAACTTGACTACTCACGGCGCCACTAACGGGCTGACGGGCC

ATCCGTCTTCCGGCGTGCGACTGTGCAACATGGCCTGCTCGGGTCTCGGCGTGGACCCTAGGGCGCTGAA

AGTGGACACTGTTTGGCGGCACCTGCGCGTGTGCTCTCATTGTTGACGTATGGTGAGGGTATTGTCAAAT

CGCACGGTTCGACAACAAGCGTACCGTCGAGTTTGGTGCAATCGGATGCCTACTACCATGGGCGGAGCCG

GCGTGCATTCAACACTCGACGTCCTGTATCAACCGGATGCCAACTTGGTTGGTGGTGCCGGCGCAAACAG

GACACTGAATCGATCTTGGTGGTACAACCCACATGTTGGTGGTCAAGTTGGTGGTGTAACTATCGGAGTC

AAAACATGATGGCGGCGATGGACGCCACAGCACTCACCCCCTACGTCCCTCGCTGAGTGTATCGTGTGTT

ATCCATCCATACACATACCTGTTTGAGCTGTGCGTTGAACACAAGAGGATGAGAGTTGCCAAACACACAG

ACCACACTCCAGT

>LK-B23

CCTACTAGGTACTTCGATTTTCCTATAATTAGACTACAGACGGGCGCCACTAATGGGCTGACGGGTTATCCGTCGTCTGGCGTGCGACTGTGCAGCATGGCGTGCTCGGGTCTCGGCGTGGACCCTTGGGCGCTGAAAGTGGATACTCTGTTTGAGCGGCACCTTTGCGTGTGCTCTCCTAAGTGTCGACGTATGGTGAGGGTAGTGTCAAGCCGCACGGTGCGACAACACAAGCGTACTGTCGAGTTTGGTGCAATCGGATGCCTACTACCATGGGCGGTGCCGGCGTGCATTCAACACTCGACGTGTGCGTCCTGTATCAACCGGATGCCAACTGC--TGTCAGTTGGTGGTGTCGGCGCAGACAGGACGCGCGCGTACGCTTGAGTCGTGTAACGCGTGCGACCCATACACGTACCTGCTTGAGCTATGCGTTGCGAGTTGGAGAGTTGCCAGACGGAGGAAATACCACAACTACTCCAGT

>LK-B10

CCTACTAGGTACTTCGATTTTCCTATAATTAGACTACAGACGGGCGCCACTAATGGGCTGACGGGTTATCCGTCGTCTGGCGTGCGACTGTGCAGCATGGCGTGCTCGGGTCTCGGCGTGGACCCTTGGGCGCTGAAAGTGGATACTCTGTTTGAGCGGCACCTTTGCGTGTGCTCTCCTAAGTGTCGACGTATGGTGAGGGTAGTGTCAAGCCGCACGGTGCGACAACACAAGCGTACTGTCGAGTTTGGTGCAATCGGATGCCTACTACCATGGGCGGTGCCGGCGTGCATTCAACACTCGACGTGTGCGTCCTGTATCAACCGGATGCCAACTGC--TGTCAGTTGGTGGTGTCGGCGCAGACAGGACGCGCGCGTACGCTTGAGTCGTGTAACGCGTGCGACCCATACACGTACCTGCTTGAGCTATGCGTTGCGAGTTGGAGAGTTGCCAGACGGAGGAAATACCACAACTACTCCAGT

>LK-B24

CCTACTAGGTACTTCGATTTTCCTATAATTAGACTACAGACGGGCGCCACTAATGGGCTGACGGGTTATCCGTCGTCTGGCGTGCGACTGTGCAGCATGGCGTGCTCGGGTCTCGGCGTGGACCCTTGGGCGCTGAAAGTGGATACTCTGTTTGAGCGGCACCTTTGCGTGTGCTCTCCTAAGTGTCGACGTATGGTGAGGGTAGTGTCAAGCCGCACGGTGCGACAACACAAGCGTACTGTCGAGTTTGGTGCAATCGGATGCCTACTACCATGGGCGGTGCCGGCGTGCATTCAACACTCGACGTGTGCGTCCTGTATCAACCGGATGCCAACTGC--TGTCAGTTGGTGGTGTCGGCGCAGACAGGACGCGCGCGTACGCTTGAGTCGTGTAACGCGTGCGACCCATACACGTACCTGCTTGAGCTATGCGTTGCGAGTTGGAGAGTTGCCAGACGGAGGAAATACCACAACTACTCCAGT

>LK-B16

CCTACTAGGTACTTCGATTTTCCTATAATTAGACTACAGACGGGCGCCACTAATGGGCTGACGGGTTATCCGTCGTCTGGCGTGCGACTGTGCAGCATGGCGTGCTCGGGTCTCGGCGTGGACCCTTGGGCGCTGAAAGTGGATACTCTGTTTGAGCGGCACCTTTGCGTGTGCTCTCCTAAGTGTCGACGTATGGTGAGGGTAGTGTCAAGCCGCACGGTGCGACAACACAAGCGTACTGTCGAGTTTGGTGCAATCGGATGCCTACTACCATGGGCGGTGCCGGCGTGCATTCAACACTCGACGTGTGCGTCCTGTATCAACCGGATGCCAACTGC--TGTCAGTTGGTGGTGTCGGCGCAGACAGGACGCGCGCGTACGCTTGAGTCGTGTAACGCGTGCGACCCATACACGTACCTGCTTGAGCTATGCGTTGCGAGTTGGAGAGTTGCCAGACGGAGGAAATACCACAACTACTCCAGT

>LK-B22

CCTACTAGGTACTTCGATTTTCCTATAATTAGACTACAGACGGGCGCCACTAATGGGCTGACGGGTTATCCGTCGTCTGGCGTGCGACTGTGCAGCATGGCGTGCTCGGGTCTCGGCGTGGACCCTTGGGCGCTGAAAGTGGATACTCTGTTTGAGCGGCACCTTTGCGTGTGCTCTCCTAAGTGTCGACGTATGGTGAGGGTAGTGTCAAGCCGCACGGTGCGACAACACAAGCGTACTGTCGAGTTTGGTGCAATCGGATGCCTACTACCATGGGCGGTGCCGGCGTGCATTCAACACTCGACGTGTGCGTCCTGTATCAACCGGATGCCAACTGC--TGTCAGTTGGTGGTGTCGGCGCAGACAGGACGCGCGCGTACGCTTGAGTCGTGTAACGCGTGCGACCCATACACGTACCTGCTTGAGCTATGCGTTGCGAGTTGGAGAGTTGCCAGACGGAGGAAATACCACAACTACTCCAGT

>LK-B31

CCTACTAGGTACTTCGATTTTCCTATAATTAGACTACAGACGGGCGCCACTAATGGGCTGACGGGTTATCCGTCGTCTGGCGTGCGACTGTGCAGCATGGCGTGCTCGGGTCTCGGCGTGGACCCTTGGGCGCTGAAAGTGGATACTCTGTTTGAGCGGCACCTTTGCGTGTGCTCTCCTAAGTGTCGACGTATGGTGAGGGTAGTGTCAAGCCGCACGGTGCGACAACACAAGCGTACTGTCGAGTTTGGTGCAATCGGATGCCTACTACCATGGGCGGTGCCGGCGTGCATTCAACACTCGACGTGTGCGTCCTGTATCAACCGGATGCCAACTGC--TGTCAGTTGGTGGTGTCGGCGCAGACAGGACGCGCGCGTACGCTTGAGTCGTGTAACGCGTGCGACCCATACACGTACCTGCTTGAGCTATGCGTTGCGAGTTGGAGAGTTGCCAGACGGAGGAAATACCACAACTACTCCAGT

>LK-B11

CCTACTAGGTACTTCGATTTTCCTATAATTAGACTACAGACGGGCGCCACTAATGGGCTGACGGGTTATCCGTCGTCTGGCGTGCGACTGTGCAGCATGGCGTGCTCGGGTCTCGGCGTGGACCCTTGGGCGCTGAAAGTGGATACTCTGTTTGAGCGGCACCTTTGCGTGTGCTCTCCTAAGTGTCGACGTATGGTGAGGGTAGTGTCAAGCCGCACGGTGCGACAACACAAGCGTACTGTCGAGTTTGGTGCAATCGGATGCCTACTACCATGGGCGGTGCCGGCGTGCATTCAACACTCGACGTGTGCGTCCTGTATCAACCGGATGCCAACTGC--TGTCAGTTGGTGGTGTCGGCGCAGACAGGACGCGCGCGTACGCTTGAGTCGTGTAACGCGTGCGACCCATACACGTACCT

>LK-B6

CCTACTAGGTACTTCGATTTTCCTATAATTAGACTACAGACGGGCGCCACTAATGGGCTGACGGGTTATCCGTCGTCTGGCGTGCGACTGTGCAGCATGGCGTGCTCGGGTCTCGGCGTGGACCCTTGGGCGCTGAAAGTGGATACTCTGTTTGAGCGGCACCTTTGCGTGTGCTCTCCTAAGTGTCGACGTATGGTGAGGGTAGTGTCAAGCCGCACGGTGCGACAACACAAGCGTACTGTCGAGTTTGGTGCAATCGGATGCCTACTACCATGGGCGGTGCCGGCGTGCATTCAACACTCGACGTGTGCGTCCTGTATCAACCGGATGCCAACTGC--TGTCAGTTGGTGGTGTCGGCGCAGACAGGACGCGCGCGTACGCTTGAGTCGTGTAACGCGTGCGACCCATACACGTACCTGCTTGAGCTATGCGTTGCGN

>LK-B7

CCTACTAGGTACTTCGATTTTCCTATAATTAGACTACAGACGGGCGCCACTAATGGGCTGACGGGTTATCCGTCGTCTGGCGTGCGACTGTGCAGCATGGCGTGCTCGGGTCTCGGCGTGGACCCTTGGGCGCTGAAAGTGGATACTCTGTTTGAGCGGCACCTTTGCGTGTGCTCTCCTAAGTGTCGACGTATGGTGAGGGTAGTGTCAAGCCGCACGGTGCGACAACACAAGCGTACTGTCGAGTTTGGTGCAATCGGATGCCTACTACCATGGGCGGTGCCGGCGTGCATTCAACACTCGACGTGTGCGTCCTGTATCAACCGGATGCCAACTGC--TGTCAGTTGGTGGTGTCGGCGCAGACAGGACGCGCGCGTACGCTTGAGTCGTGTAACGCGTGCGACCCATACACGTACCTGCTTGAGCTATGCGTTGCGAGTTGGAGAGTTGCCAGACGGAGGAAATACCACAACTACTCCAGT

>LK-B9

CCTACTAGGTACTTCGATTTTCCTATAATTAGACTACAGACGGGCGCCACTAATGGGCTGACGGGTTATCCGTCGTCTGGCGTGCGACTGTGCAGCATGGCGTGCTCGGGTCTCGGCGTGGACCCTTGGGCGCTGAAAGTGGATACTCTGTTTGAGCGGCACCTTTGCGTGTGCTCTCCTAAGTGTCGACGTATGGTGAGGGTAGTGTCAAGCCGCACGGTGCGACAACACAAGCGTACTGTCGAGTTTGGTGCAATCGGATGCCTACTACCATGGGCGGTGCCGGCGTGCATTCAACACTCGACGTGTGCGTCCTGTATCAACCGGATGCCAACTGC--TGTCAGTTGGTGGTGTCGGCGCAGACAGGACGCGCGCGTACGCTTGAGTCGTGTAACGCGTGCGACCCATACACGTACCTGCTTGAGCTATGCGTTGCGAGTTGGAGAGTTGCCAGACGGAGGAAATACCACAACTACTCCAGT

>AY691517.1 Sund Andaman IN

CCTACTAGGTACTTCGATTTTCCTATAATTAGACTACAGACGGGCGCCACTAACGGGCTGACGGGCTATCCGTCGTCTGGCGTGCGACTGTGCAGCATGGCGTGCTCGGGTCTCGGCGTGGACCCTTGGGCGCTGAAAGTGGATACTCTGTTTGAGCGGCACCTTTGCGTGTGCTCTCCTAAGTGTCGACGTATGGTGAGGGTAGTGTCAAGCCGCACGGTGCGACAACACAAGCGTACTGTCGAGTTTGGTGCAATCGGATGCCTACTACCATGGGCGGTGCCGGCGTGCATTCAACACTCGACGTGCGTGTCCTGTATCAACCGGATGCCAACTGCCGTGTCAGTTGGTGGTGTCGGCGCAGACAGGACGCGCGCGTACGCTTGAGTCGTGTAACGCGTGCGACCCATACACGTACCTGCTTGAGCTGTGCGTTGCGAGCTGGAGAGTTGCCAGACGGAGCAAATACCACATACTCCA

>AF369562.1 Sund Sarawak2 MY

CCTACTAGGTACTTCGATTTTCCTATAATTAGACTACAGACGGGCGCCACTAACGGGCTGACGGGCTATC

CGTCGTCTGGCGTGCGACTGTGCAGCATGGCGTGCTCGGGTCTCGGCGTGGACCCTTGGGCGCTGAAAGT

GGATACTCTGTTTGAGCGGCACCTTTGCGTGTGCTCTCCTAAGTGTCGACGTATGGTGAGGGTAGTGTCA

AGCCGCACGGTGCGACAACACAAGCGTACTGTCGAGTTTGGTGCAATCGGATGCCTACTACCATGGGCGG

TGCCGGCGTGCATTCAACACTCGACGTGCGTGTCCTGTATCAACCGGATGCCAACTGCCGTGTCAGTTGG

TGGTGTCGGCGCAGACAGGACGCGCGCGTACGCTTGAGTCGTGTAACTCGTGCGACCCATACACGTACCT

GCTTGAGCTGTGCGTTGCGAGCTGGAGAGTTGCCAGACGGAGAAATACCACATACTCCAGT

>AF469857.1 Sund Thailand

CCTACTAGGTACTTCGATTTTCCTATAATTAGACTACAGACGGGCGCCACTAACGGGCTGACGGGCTATC

CGTCGTCTGGCGTGCGACTGTGCAGCATGGCGTGCTCGGGTCTCGGCGTGGACCCTTGGGCGCTGAAAGT

GGATACTCTGTTTGAGCGGCACCTTTGCGTGTGCTCTCCTAAGTGTCGACGTATGGTGAGGGTAGTGTCA

AGCCGCACGGTGCGACAACACAAGCGTACTGTCGAGTTTGGTGCAATCGGATGCCTACTACCATGGGCGG

TGCCGGCGTGCATTCAACACTCGACGTGCGTGTCCTGTATCAACCGGATGCCAACTGCTGTGTCAGTTGG

TGGTGTCGGCGCAGACAGGACGCGCGCGTACGCTTGAGTCGTGTAACGCGTGCGACCCATACACGTACCT

GCTTGAGCTGTGCGTTGCGAGCTGGAGAGTTGCCAGACGGAGCAAATACCACATACTCCAGT

>GQ480823.1 VagusB E Timor

CCTACTAGGTACTTCGATTTTCCTATAATTAGACTACAGACGGGCGCCACTAACGGGCTG

ACGGGCTATCCGTCGTCTGGCGTGCGACTGTGCAGCATGGCGTGCTCGGGTCTCGGCGTGGACCCTTGGG

CGCTGAAAGTGGATACTCTGTTTGAGCGGCACCTTTGCGTGTGCTCTCCTAAGTGTCGACGTATGGTGAG

GGTAGTGTCAAGCCGCACGGTGCGACAACACAAGCGTACTGTCGAGTTTGGTGCAATCGGATGCCTACTA

CCATGGGCGGTGCCGGCGTGCATTCAACACTCGACGTGCGTGTCCTGTATCAACCGGATGCCAACTGCCG

TGTCAGTTGGTGGTGTCGGCGCAGACAGGACGCGCGCGTACGCTTGAGTCGTGTAACTCGTGCGACCCAT

ACACGTACCTGCTTGAGCTGTGCGTTGCGAGCTGGAGAGTTGCCAGACGGAGCAAATACCACATACTCCA

GT

>AY662258.1 Sund Sarawak1 MY

CCTACTAGGTACTTCGATTTTCCTATAATTAGACTACAGACGGGCGCCACTAACGGGCTGACGGGCTATC

CGTCGTCTGGCGTGCGACTGTGCAGCATGGCGTGCTCGGGTCTCGGCGTGGACCCTTGGGCGCTGAAAGT

GGATACTCTGTTTGAGCGGCACCTTTGCGTGTGCTCTCCTAAGTGTCGACGTATGGTGAGGGTAGTGTCA

AGCCGCACGGTGCGACAACACAAGCGTACTGTCGAGTTTGGTGCAATCGGATGCCTACTACCATGGGCGG

TGCCGGCGTGCATTCAACACTCGACGTGCGTGTCCTGTATCAACCGGATGCCAACTGCCGTGTCAGTTGG

TGGTGTCGGCGCAGACAGGACGCGCGCGTACGCTTGAGTCGTGTAACTCGTGCGACCCATACACGTACCT

GCTTGAGCTGTGCGTTGCGAGCTGGAGAGTTGCCAGACGGAGAAATACCACATACTCCAGT

>AY662445.1 Epir Vietnam

CCTACTAGGTACTTCGATTTTCCTATAATTAGACTACAGACGGGCGCCACTAACGGGCTGACGGGCTATC

CGTCGTCTGGCGTGCGACTGTGCAGCATGGCGTGCTCGGGTCTCGGCGTGGACCCTTGGGCGCTGAAAGT

GGATACTCTGTTTGAGCGGCACCTTTGCGTGTGCTCTCCTAAGTGTCGACGTATGGTGAGGGTAGTGTCA

AGCCGCACGGTGCGACAACACAAGCGTACTGTCGAGTTTGGTGCAATCGGATGCCTACTACCATGGGCGG

TGCCGGCGTGCATTCAACACTCGACGTGCGTGTCCTGTATCAACCGGATGCCAACTGCTGTGTCAGTTGG

TGGTGTCGGCGCAGACAGGACGCGCGCGTACGCTTGAGTCGTGTAACGCGTGCGACCCATACACGTACCT

GCTTGAGCTGTGCGTTGCGAGCTGGAGAGTTGCCAGACGGAGCAAATACCACATACTCCAGT

>GQ480826.1 Sund E Timor

CCTACTAGGTACTTCGATTTTCCTATACTTAGACTACAGACGGGCGCCACTAACGGGCTG

ACGGGTTATCCGTCGTCTGGCGTGCGACTGTGCAGCATGGCGTGCTCGGGTCTCGGCGTGGACCCTTGGG

CGCTGAAAGTGGATACTCTGTTTGAGCGGCACCTTTGCGTGTGCTCTCCTAAGTGTCGACGTATGGTGAG

GGTAGTGTCAAGCCGCACGGTGCGACAACACAAGCGTACTGTCGAGTTTGGTGCAATCGGATGCCTACTA

CCATGGGCGGTGCCGGCGTGCATTAAACACTCGACGTGCGTGTCCTGTATCAACCGGATGCCAACTGCTG

TGTCAGTTGGTGGTGTCGGCGCAGACAGGACGCGCGCGTACGCTTGAGTCGTGTAACGCGTGCGACCCAT

ACACGTACCTGCTTGAGCTATGCGTTGCGAGTTGGAGAGTTGCCAGACGGAGGAGATACCACAAACACTC

CAGT

>FJ457631.1 Vagus China

CCTACTAGGTACTGAGATTTAACTATGACTTGACTACAGACGGCGCCACTAAA

GGGCTGACGGGCCATCCGTCGTCCGGCGTGCGACTGTGCAGCATGGCGTGCTCGGGTCTCGGCGTGGACC

CTTGGGCGCTGAAAGTGGACACTGTTTGGCGGCACCTGCGCGTGTGCTCTCAGTGTTGACGTATGGTGAG

GGTAGTGTCAAGTCGCACGGTTCGACAACAAGCGTACCGTCGAGTTTGGTGCAATCGGATGCCTACTACC

ATGGGCGGTGCCGGCGTGCATTCAACACACTCGACGTCCCGTACCAACCGGATGCCTGTGAAGGCGGTGC

CGGCGCAGACGGGACACTGAATTGATCTTGGTGATATTGGGGGATGATGGATGATGTGTGTCGCGAGTGA

CAACCGGATGCCAGCGATGGCGGTGCCGGCGCACACGAGCGCTCACACACGCCTCTCCCCTCGGTCGCTT

GTGGCGTGTAACGCGTGTGATCCATACACTTACCTGTTTGAGCCGTGCGTTCGACGCAAGTATGAGAGTT

GCCAGACAAACCACATTCCAGT

>FJ654649.1 Vagus Indonesia

CCTACTAGGTACTGAGATTTAACTATGACTTGACTACAGACGSCGCCACTAAAGGGCTGACGGGCCATCC

GTCGTCCGGCGTGCGACTGTGCAGCATGGCGTGCTCGGGTCTCGGCGTGGACCCTTGGGCGCTGAAAGTG

GACACTGTTTGGCGGCACCTGCGCGTGTGCTCTCAGTGTTGACGTATGGTGAGGGTAGTGTCAAGTCGCA

CGGTTCGACAACAAGCGTACCGTCGAGTTTGGTGCAATCGGATGCCTACTACCATGGGCGGTGCCGGCGT

GCATTCAACACACTCGACGTCCCGTACCAACCGGATGCCTGTGAAGGCGGTGCCGGCGCAGACGGGACAC

TGAATTGATCTTGGTGATATTGGGTGGGGGATGATGGATGATGTGTGTCGCGAGTGACAACCGGATGCCA

GCGATGGCGGTGCCGGCGCACACGAGCGCTCACACACGCCTCTCCCCTCGGTCGCTTGTGGCGTGTAACG

CGTGTGATCCATACACTTACCTGTTTGAGCCGTGCGTTCGACGCAAGTATGAGAGTTGCCAGACAAACCA

CATTCCAGT

> LK-A21 and LK-A22

CCTACTAGGTACTGAGAGATTCCTATAACTTGACTACAGACGGCGCCACAAACGGGCTGACGGGCCATCCGTCGTCCGGCGTGCGACTGTGCAGCATGGCGTGCTCGGGTCTCGGCGTGGACCCTTGGGCGCTGAAAGTGGACACTGTTTGGCGGCACCTGCGCGTGTGCTCTCAGTGTTGACGTATGGTGAGGGTAGTGTCAAATCGCACGGTTCGACAACAAGCGTACCGTCGAGTTTGGTGCAATCGGATGCCTACTACCATGGGCGGAGCCGGCGTGCATTCAACACTCGACGTCCTGTATCAACCGGATGCCAACTTGGTTGGTGGTGCCGGCGCAGACAGGACACTGAATCGATCTTGGTGGTACAACCCACATGTGGGT
