# Supplementary material for: Genetic evidence for malaria vectors of the Anopheles sundaicus complex in Sri Lanka with morphological characteristics attributed to Anopheles subpictus species B
Source: Malar J. 2010 Nov 29;9:343. doi: 10.1186/1475-2875-9-343 (PMC3009661; doi:10.1186/1475-2875-9-343)
Supplement: Additional File 4 — Morphological differentiation of An. sundaicus, An. vagus, An. pseudosundaicus and An. subpictus. Reported morphological characteristics that differentiate An. sundaicus, An. vagus, An. pseudosundaicus and An. subpictus populations and their distribution among sibling species of the Subpictus Complex [file 1475-2875-9-343-S4.DOC]

**Morphological differentiation of *An. sundaicus*, *An. vagus, An. pseudosundaicus* and *An. subpictus***

| **Characters** | ***An. sundaicus*** [6, 31] | ***An. vagus*** [6] | ***An. pseudosundaicus*** [31] | | ***An. subpictus* *s.l.*** [6, 31] | ***An. subpictus* sibling species** | | | | |
| --- | --- | --- | --- | --- | --- | --- | --- | --- | --- | --- |
|  |  |  |  |  |  | **A** [7] | **B** [7] | **C** [7] | **D** [7] | |
| Palpi | Apical pale band equal to or little longer than subapical dark band | Subapical pale band ¼ length of apical pale band | Apical pale band shorter than subapical dark band | | Apical pale band longer than or equal to subapical dark band | Apical pale band longer than subapical dark band | Apical pale band shorter than subapical dark band | Apical pale band equal to subapical dark band | | |
| Wing | Prehumeral dark spot complete and preapical dark spot on costa long | | | | Prehumeral dark spot usually incomplete and preapical dark spot on costa short | Variations not reported | | | | |
| Leg | Femur and tibia speckled | Femur and tibia not speckled | | | | | | | | |
| Larval seta 4M | 3 branches | Not reported | | Single branch (rarely 2 branches) | 2 branches (rarely 3 branches) | 2 branches | | 3 branches | | |
| Pupal seta 7-1 | 3 branched | 2 branched | | 2-5(3) branched | Simple as long as setae 6 and 9 | Simple as long as setae 6 and 9 | 4-5 branched; shorter than setae 6 and 9 | 2branched | | 3 branched |
| Egg (number of float ridges) | 20 | 20 - 30 | | Not reported | 30 - 40 | 31 - 36 | 16 – 20 | 25 - 29 | | 21 - 24 |
