# Supplementary material for: Genetic evidence for malaria vectors of the Anopheles sundaicus complex in Sri Lanka with morphological characteristics attributed to Anopheles subpictus species B
Source: Malar J. 2010 Nov 29;9:343. doi: 10.1186/1475-2875-9-343 (PMC3009661; doi:10.1186/1475-2875-9-343)
Supplement: Supplementary file 4 [file 1475-2875-9-343-S2.DOC]

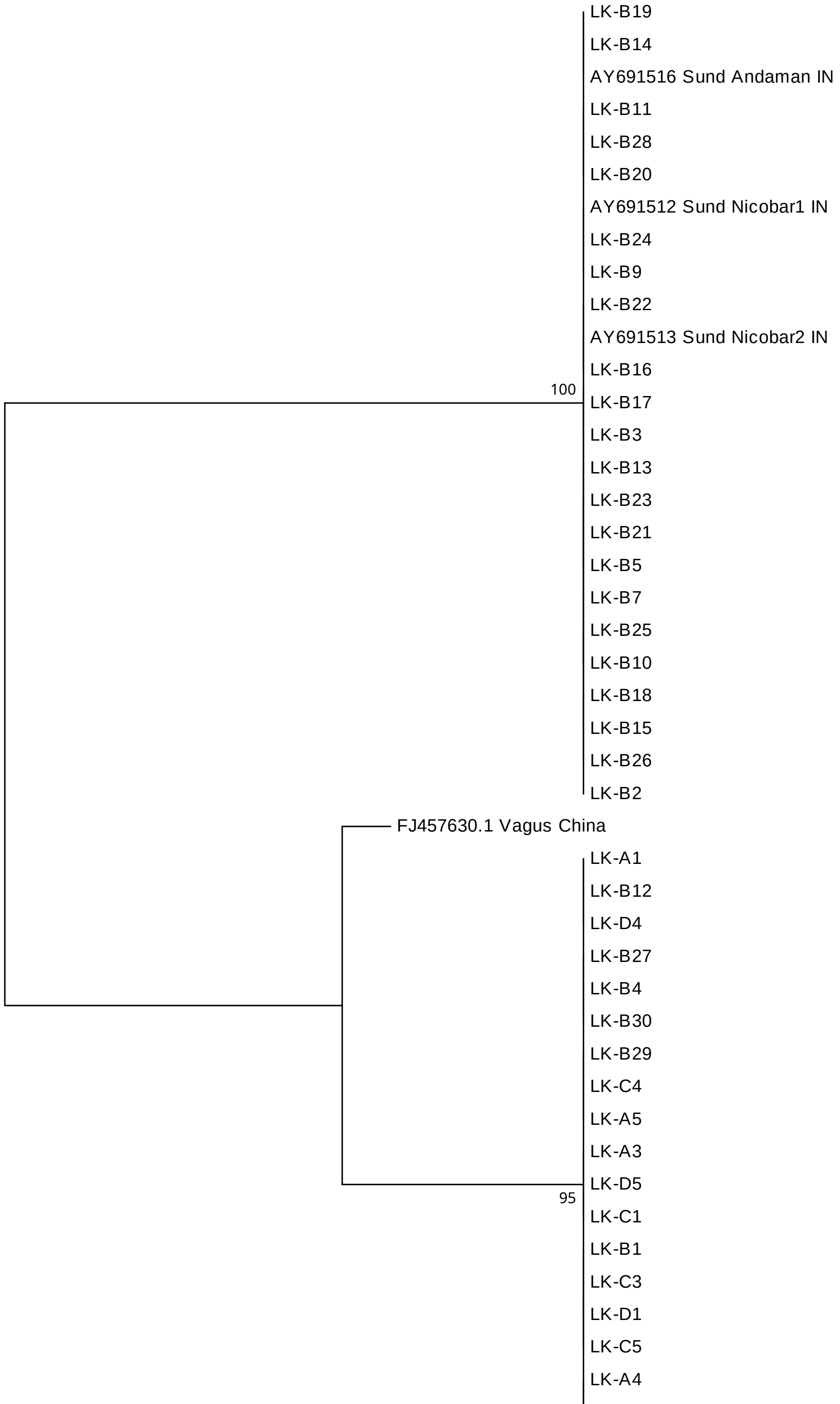
LK-B19
LK-B14
AY691516 Sund Andaman IN
LK-B11
LK-B28
LK-B20
AY691512 Sund Nicobar1 IN
LK-B24
LK-B9
LK-B22
AY691513 Sund Nicobar2 IN
LK-B16
100
LK-B17
LK-B3
LK-B13
LK-B23
LK-B21
LK-B5
LK-B7
LK-B25
LK-B10
LK-B18
LK-B15
LK-B26
LK-B2
FJ457630.1 Vagus China
LK-A1
LK-B12
LK-D4
LK-B27
LK-B4
LK-B30
LK-B29
LK-C4
LK-A5
LK-A3
LK-D5
95
LK-C1
LK-B1
LK-C3
LK-D1
LK-C5
LK-A4
LK-D2
LK-C2
LK-A2
LK-B8
0.005
